# Purine salvage–associated metabolites as biomarkers for early diagnosis of esophageal squamous cell carcinoma: a diagnostic model–based study

**DOI:** 10.1038/s41420-024-01896-6

**Published:** 2024-03-14

**Authors:** Yawen Sun, Wenjuan Liu, Mu Su, Tao Zhang, Xia Li, Wenbin Liu, Yuping Cai, Deli Zhao, Ming Yang, Zhengjiang Zhu, Jialin Wang, Jinming Yu

**Affiliations:** 1 Department of Medical Epidemiology and Biostatistics, Shandong Cancer Hospital and Institute, Shandong First Medical University and Shandong Academy of Medical Sciences, Jinan, Shandong 250117 China; 2 Shandong Provincial Key Laboratory of Radiation Oncology, Cancer Research Center, Shandong Cancer Hospital and Institute, Shandong First Medical University and Shandong Academy of Medical Sciences, Jinan, Shandong 250117 China; 3 Berry Oncology Corporation, Beijing, 102206 China; 4 Department of Biostatistics, School of Public Health, Cheeloo College of Medicine, Shandong University, Jinan, Shandong 250012 China; 5 Department of Public Health, Shandong Public Health Clinical Center, Shandong University, Jinan, Shandong 250013 China; 6 Interdisciplinary Research Center on Biology and Chemistry, Shanghai Institute of Organic Chemistry, Chinese Academy of Sciences, Shanghai, 200032 China; 7 Tumor Preventative and Therapeutic Base of Shandong Province, Feicheng People’s Hospital, Feicheng, Shandong 271600 China; 8 Shandong Provincial Key Laboratory of Radiation Oncology, Cancer Research Center, Shandong Cancer Hospital and Institute, Jinan, Shandong 250117 China; 9 Department of Radiation Oncology and Shandong Provincial Key Laboratory of Radiation Oncology, Shandong Cancer Hospital and Institute, Shandong First Medical University and Shandong Academy of Medical Sciences, Jinan, Shandong 250117 China

**Keywords:** Diagnostic markers, Oesophageal cancer, Cancer screening, Cancer prevention, Cancer metabolism

## Abstract

Esophageal squamous cell carcinoma (ESCC) remains an important health concern in developing countries. Patients with advanced ESCC have a poor prognosis and survival rate, and achieving early diagnosis remains a challenge. Metabolic biomarkers are gradually gaining attention as early diagnostic biomarkers. Hence, this multicenter study comprehensively evaluated metabolism dysregulation in ESCC through an integrated research strategy to identify key metabolite biomarkers of ESCC. First, the metabolic profiles were examined in tissue and serum samples from the discovery cohort (*n* = 162; ESCC patients, *n* = 81; healthy volunteers, *n* = 81), and ESCC tissue-induced metabolite alterations were observed in the serum. Afterward, RNA sequencing of tissue samples (*n* = 46) was performed, followed by an integrated analysis of metabolomics and transcriptomics. The potential biomarkers for ESCC were further identified by censoring gene-metabolite regulatory networks. The diagnostic value of the identified biomarkers was validated in a validation cohort (*n* = 220), and the biological function was verified. A total of 457 dysregulated metabolites were identified in the serum, of which 36 were induced by tumor tissues. The integrated analyses revealed significant alterations in the purine salvage pathway, wherein the abundance of hypoxanthine/xanthine exhibited a positive correlation with HPRT1 expression and tumor size. A diagnostic model was developed using two purine salvage–associated metabolites. This model could accurately discriminate patients with ESCC from normal individuals, with an area under the curve (AUC) (95% confidence interval (CI): 0.680–0.843) of 0.765 in the external cohort. Hypoxanthine and HPRT1 exerted a synergistic effect in terms of promoting ESCC progression. These findings are anticipated to provide valuable support in developing novel diagnostic approaches for early ESCC and enhance our comprehension of the metabolic mechanisms underlying this disease.

## Introduction

Esophageal cancer is a malignant tumor originating from the esophageal mucosa. More than 480,000 new cases are diagnosed each year worldwide, half of which occur in China [1]. Its mortality rate ranks third among all cancers prevalent in China [2]. Additionally, it poses a huge disease and economic burden. In comparison to Western nations, the Chinese population primarily suffers from esophageal squamous cell carcinoma (ESCC) as the dominant pathological subtype, representing over 90% of cases [2]. Despite recent advancements in the diagnosis and treatment of esophageal cancer, the prognosis of patients still requires significant improvement, as evidenced by a relatively low 5-year survival rate of only 15%–25% [3, 4]. Early diagnosis (TNM: 0-II) is key to improving overall survival (OS), and the 5-year survival rate of patients can be improved to 47%–83% [3, 5, 6]. Consequently, there is a pressing need to develop a diagnostic method that is sensitive, specific, reliable, and cost-effective, particularly for population screening, to address the practical requirements of prevention and treatment of esophageal cancer. Imaging examinations such as barium meal radiography and computed tomography are highly suitable for the auxiliary diagnosis and clinical staging of patients with advanced-stage esophageal cancer [7, 8]. Notably, esophageal endoscopy together with biopsy remains the gold standard for esophageal cancer diagnosis, which has an elevated detection rate in patients with early-stage esophageal cancer [9–11]. However, this method has the disadvantages of low subject compliance, technical difficulty, and high cost; therefore, it is applied only for screening in areas with a high incidence rate of esophageal cancer (such as in Feicheng) [12]. Cytokeratin-19-fragment (CYFRA21-1), carcinoembryonic antigen (CEA), and squamous cell cancer (SCC) antigens are commonly utilized biomarkers for ESCC. However, their accuracy remains relatively low [13–15]. For population-level screening, early-stage, noninvasive diagnostic tests will allow rapid identification of high-risk groups and determine the requirement for further endoscopy.

Metabolic reprogramming is regarded as an important feature of tumors [16]. Tumor cells have a tendency to establish a perpetual pro-anabolic state of metabolism, leading to uncontrolled accumulation of transformed cells and tumor growth [17]. High-throughput metabolomics is of particular interest, as it can reflect disease-associated cellular biochemical activities that are not detected by other omics technology [18, 19]. Accumulating evidence suggests that metabolic biomarkers can help detect ESCC [20, 21]. Zhang et al. performed serum metabolomics studies using gas chromatography-mass spectrometry and reported that the combination of 2-ketoisocaproic acid, hypoxanthine, L-glutamate, and L-aspartate had reasonable performance in discriminating patients with ESCC from those with esophageal squamous dysplasia [22]. In our previous study, a serum metabolomics approach based on liquid chromatography-mass spectrometry (LC-MS) was utilized, and an increased risk of ESCC was observed, as evidenced by five metabolites (hypoxanthine, inosine, carnitine [14:1], glycochenodeoxycholate, and PC [P-18:0/18:3]) [23]. Despite the surging interest in metabolic biomarkers, only few studies have verified their availability and utility at the biological mechanism level, which may at least partially affect the practical application of biomarkers.

This study comprehensively investigated biomarkers for ESCC. Metabolic profiling was performed to assess ESCC tumor tissue-induced metabolite alterations in the serum. Moreover, an integrated analysis of metabolomics and transcriptomics was performed to visualize gene-metabolite regulatory networks to gain novel mechanistic insights into metabolic disorders and further screen for key metabolic markers of ESCC. The clinical value of the biomarkers was verified using an independent validation cohort and biological experiments.

## Results

### Result 1: Metabolic profiles in the tissue and serum revealed significantly altered metabolites in ESCC

**Fig. 1:**
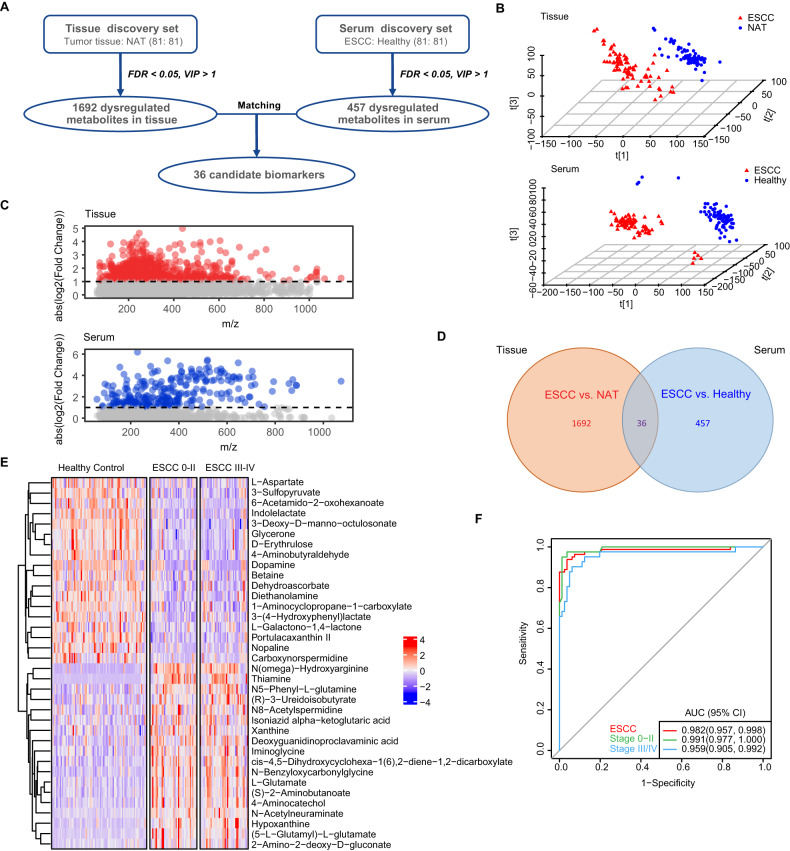
Metabolic signatures in ESCC based on metabolomics data from the discovery cohort. **A** Flowchart showing the analytical strategy to identify the dysregulated metabolites in the ESCC tissue and serum. A total of 162 participants, including 81 patients with ESCC and 81 healthy volunteers, were included in the discovery cohort. **B** Supervised PLS-DA plots for the metabolic profiles of ESCC samples and control samples. ESCC tissues vs NAT (up); serum of patients with ESCC vs serum of healthy subjects (down). **C** Metabolic dysregulation in ESCC tissue and serum samples. Distributions of absolute log2 fold-change for significantly dysregulated metabolic peaks in the ESCC tissue (up) and serum (down). Each circle in the plot represents one differential feature. Red: metabolic peaks with absolute log2 fold-change greater than 1 in the tissue set; blue: metabolic peaks with absolute log2 fold-change greater than 1 in the serum set; gray: metabolic peaks with absolute log2 fold-change less than 1. **D** Venn diagram showing the shared and unique dysregulated metabolic peaks between ESCC tissues and serum samples. **E** Heatmap showing the relative abundance of 36 biomarker candidates in healthy volunteers, patients with early-stage ESCC (TNM: 0/I/II), and patients with advanced-stage ESCC (TNM: III/IV). The colors from blue to red indicate the increase in the abundance of the metabolites. **F** ROC curves of the diagnostic performances using the combination of 36 biomarker candidates in serum from patients in the discovery cohort. Red: healthy subjects vs patients with ESCC; green: healthy subjects vs patients with early-stage ESCC (TNM: 0/I/II); blue: healthy subjects vs patients with advanced-stage ESCC (TNM: III/IV). ESCC esophageal squamous cell carcinoma, NAT normal tissues adjacent to the tumor, PLS-DA partial least squares–discriminant analysis, ROC receiver operating characteristic, TNM tumor-node-metastasis classification system (8th edition, 2017).

The metabolic profiling of paired tissue samples (from patients with ESCC (*n* = 81), tumor tissues vs normal tissues adjacent to the tumor (NAT)) and serum samples (from patients with ESCC (*n* = 81) and healthy volunteers (*n* = 81)) were conducted using LC-MS-based untargeted metabolomics. The purpose of this investigation was to explore the global metabolic dysregulation in ESCC, as illustrated in Fig. 1A. Table 1 elaborates on the features of participants in the discovery cohort. Supplementary Fig. 1 shows typical base peak chromatograms detected in the positive (ESI+) and negative (ESI−) modes. The relative standard deviation (RSD) of each ion peak was calculated in the quality control samples, and peaks with RSD > 30% were eliminated to reduce data noise. Unsupervised models of principal component analysis (PCA) depicted differences between tumor tissues and NAT as well as between serum samples from patients with ESCC and those from healthy volunteers (Supplementary Fig. 2). Subsequently, the results of supervised models of partial least squares-discriminant analysis (PLS-DA) (Fig. 1B) suggest the apparent difference between tumor tissues and NAT (up) and a significant distinction between serum samples from patients with ESCC and those from healthy volunteers (down). The good fitting of the PLS-DA models was confirmed through permutation tests, as depicted in Supplementary Fig. 3. Using the Metabolite annotation and Dysregulated Network Analysis (MetDNA) strategy, a total of 4576 metabolites in tissue samples and 1600 metabolites in serum samples were successfully annotated. After applying significance thresholds of false discovery rate (FDR)-adjusted *p* < 0.05 and a variable importance in projection (VIP) value greater than 1, a total of 1692 dysregulated metabolites in tissue samples and 457 dysregulated metabolites in serum samples were identified, as presented in Fig. 1C.

**Table 1:** Demographic and clinical characteristics of ESCC patients and healthy volunteers in discovery cohort.

Characteristics	ESCC^a^ patients	Healthy volunteers	*p* value
Total Number	81	81	
Gender (male/female)	60/21	63/18	0.713
Age (mean ± SD^b^, year)	62.49 ± 8.1	59.25 ± 7.8	0.010
BMI^c^ (mean ± SD^b^)	23.45 ± 3.2	24.61 ± 3.0	0.018
Smoker, *n* (%)	37 (45.7)	35 (43.2)	0.874
Drinker, *n* (%)	35 (43.2)	32 (39.5)	0.750
TNM^d^, *n* (%)			
0/I	5 (6.2)	—	
II	38 (46.9)	—	
III	32 (39.5)	—	
IV	6 (7.4)	—	
Clinical T stage, *n* (%)		—	
T0–T2	18 (22.2)	—	
T3–T4	63 (77.8)	—	
Clinical *N* stage, *n* (%)		—	
N0	43 (53.1)	—	
N1–N3	38 (46.9)	—	
Tumor size, *n* (%)		—	
<5 cm	35 (43.2)	—	
≥5 cm	46 (56.8)	—	
Tumor location, *n* (%)		—	
Upper thoracic	3 (3.7)	—	
Middle thoracic	59 (72.8)	—	
Lower thoracic	19 (23.5)	—	
Differentiation grade, *n* (%)		—	
G1	20 (25.0)	—	
G2	35 (43.8)	—	
G3	25 (31.2)	—	

^a^*ESCC* esophageal squamous cell carcinoma.

^b^*SD* standard deviation.

^c^*BMI* body mass index.

^d^*TNM* tumor-node-metastasis classification system (8th edition, 2017).

Further analysis of the metabolic alterations shared in ESCC tissues and serum was conducted. The metabolites from two data sets were matched according to polarity, the mass-to-charge ratio (m/z ± 30 ppm), and retention time (±30 s). The resulting data indicated 36 shared dysregulated metabolites between the two sample types (Fig. 1D). Detailed information on these metabolites is listed in Supplementary Table 1. A heatmap (Fig. 1E) displays the relative levels of these 36 metabolites in the serum samples of patients with ESCC and healthy volunteers. Serum metabolites that were potential candidate biomarkers were identified to construct a random Forest diagnostic model, with fivefold cross-validation. Receiver operating characteristic (ROC) curves indicated that the model provided feasibility in effectively differentiating affected individuals from the normal controls (area under the curve (AUC) = 0.982, 95% confidence interval (CI): 0.957–0.998; Fig. 1F). Importantly, the model had an excellent diagnostic performance for early-stage (TNM: 0/I/II) ESCC (AUC = 0.991, 95% CI: 0.977–1.000), with the sensitivity recorded to be 97.5% and specificity to be 98.9% (Fig. 1F).

### Result 2: Pathway analysis by integrating metabolomics and transcriptomics identified critical gene-metabolite alterations and ESCC biomarkers

**Fig. 2:**
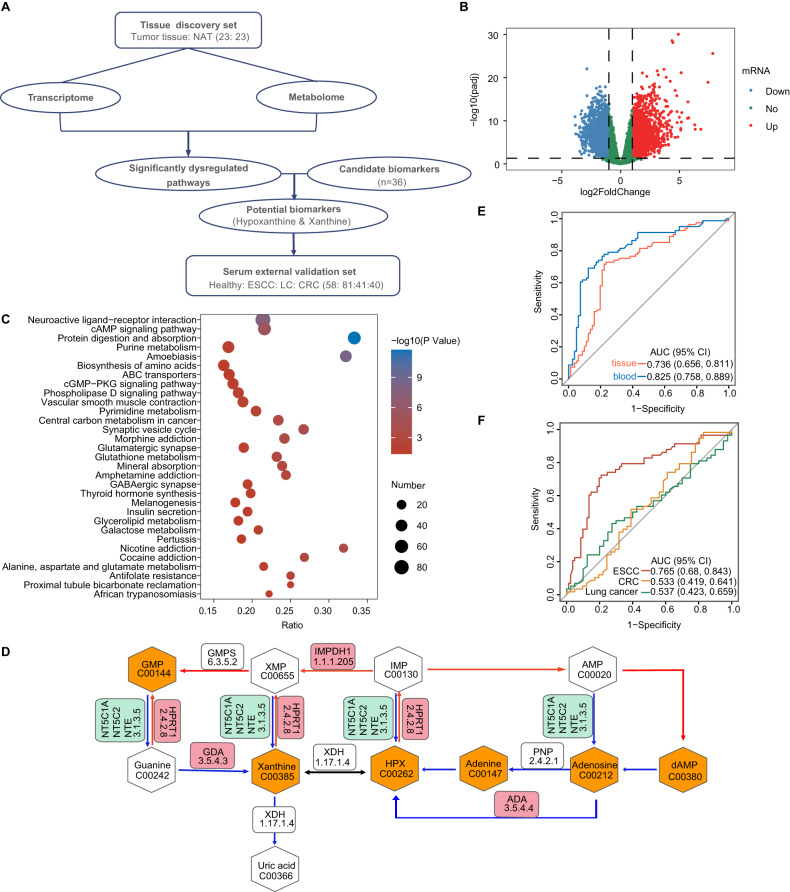
Integrated analyses of metabolomics and transcriptomics data showing the critical metabolic dysregulations of patients with ESCC. **A** Diagram showing the procedure for the integrated analysis of tissue metabolome and transcriptome in the discovery cohort. Untargeted metabolic detection and transcriptome sequencing were carried out for paired tissues from 23 patients with ESCC. **B** Volcano plot showing the DEGs in the tumor tissue. **C** Enriched KEGG pathways identified in the hypergeometric distribution test for DEGs and metabolites. **D** Dysregulated purine salvage pathway in patients with ESCC. Synchronous upregulated expression of the gene (HPRT1) and its metabolic substrates (hypoxanthine and xanthine) in patients with ESCC, leading to enhanced synthesis of guanosine monophosphate. **E** ROC curves showing the diagnostic performance of the combination of two potential biomarkers (hypoxanthine and xanthine) in the discovery cohort. Red: tumor tissues vs NAT; blue: serum of healthy subjects vs serum of patients with ESCC. **F** ROC curves showing the diagnostic performances of the combination of two potential biomarkers (hypoxanthine and xanthine) in the external validation cohort. Red: serum of healthy subjects vs serum of patients with ESCC; yellow: serum of healthy subjects vs patients with colorectal cancer; green: serum of healthy subjects vs patients with lung cancer. DEGs differentially expressed genes, ESCC esophageal squamous cell carcinoma, KEGG Kyoto Encyclopedia of Genes and Genomes, ROC receiver operating characteristic, NAT normal tissues adjacent to the tumor.

As metabolite changes may not be entirely related to disease phenotypes, further exploration of the biological background of metabolites was needed to target more reliable biomarkers. A comprehensive transcriptome–metabolome analysis was performed on paired tissues from patients with ESCC (Fig. 2A). RNA sequencing of 46 tissue samples was performed, and the results of the volcano plot presented significant differences in gene expression between tumor tissues and NAT (Fig. 2B). In total, 3110 differentially expressed mRNAs were identified as per the criteria of |log2(foldchange)| > 1 and FDR-adjusted *p* < 0.05. To visualize the gene-metabolite network in ESCC, pathway enrichment analysis of differentially expressed genes (DEGs) and dysregulated metabolites was performed using the hypergeometric distribution test. The data indicated remarkable alterations in 31 pathways (Fig. 2C and Supplementary Table 2), including the cAMP signaling pathway, neuroactive ligand–receptor interaction, protein digestion, absorption, purine metabolism, etc.

In the altered pathways, metabolites and genes were labeled as upregulated and downregulated according to the fold change to visualize metabolic reprogramming in patients with ESCC. For the transcriptome, log2(foldchange) > 1 indicates genes with high expression, and log2(foldchange) < −1 indicates genes with low expression. For the metabolome, foldchange > 1.2 indicates upregulated metabolites, and foldchange < 0.83 indicates downregulated metabolites. After review of all 31 pathways, we found that the changes in the purine metabolism pathway are the most biologically interpretable, hence being the focus of further research. A total of 38 metabolites and genes displayed varying degrees of dysregulation. Notably, a substantial number of genes and metabolites in the purine salvage pathway exhibited significant alteration, while few alterations were observed in the metabolites associated with purine de novo synthesis and purine catabolism. Particularly, synchronized gene–metabolite alterations were observed in the purine salvage pathway of patients with ESCC (Fig. 2D). When compared to NAT, tumor tissues of ESCC exhibited significantly abundant expression in the metabolites, including hypoxanthine, xanthine, adenine, adenosine, etc. Some genes had significantly upregulated expression, including HPRT1, IMPDH1, GDA, ADA, etc. The downstream metabolites guanosine monophosphate and deoxyadenosine monophosphate had significantly increased levels. Remarkably, the data confirmed that hypoxanthine and xanthine are serum metabolites with significant alterations induced by the ESCC tumor tissue. The homeostasis of both purine metabolites is mediated by hypoxanthine-guanine phosphoribosyl transferase (HGPRT), a key metabolic enzyme encoded by HPRT1 in the purine salvage pathway (Fig. 2D).

Consequently, based on hypoxanthine and xanthine, the Random Forest model was re-established for ESCC diagnosis, with a fivefold cross-validation. In the discovery cohort, ROC curves exhibited strong diagnostic performance for the metabolite biomarkers in the serum dataset, with an AUC value of 0.825 (95% CI: 0.758–0.889), as illustrated in Fig. 2E. To further verify their diagnostic potential, the efficacy of the model was evaluated in an external validation cohort, with the details enlisted (*n* = 220) in Supplementary Table 3. The results from ROC curves showed that the model performed well in distinguishing patients with ESCC from normal individuals, with an AUC value of 0.765 (95% CI: 0.680–0.843; Fig. 2F). However, this model could barely distinguish normal individuals from patients with lung or colorectal cancer, with AUC values of 0.537 (95% CI: 0.423–0.659) and 0.533 (95% CI: 0.419–0.641), respectively (Fig. 2F). These results suggest that hypoxanthine and xanthine have great potential as biomarkers for ESCC detection. These outcomes reveal that upregulated expression of the hypoxanthine/xanthine-HPRT1 network may closely indicate ESCC development. Elucidating the biological role of this network in ESCC may be helpful in clarifying the diagnostic value of the biomarkers, which is the premise and the basis for their clinical application.

### Result 3: The network of hypoxanthine/xanthine-HPRT1 was upregulated in ESCC and linked to malignant characteristics

**Fig. 3:**
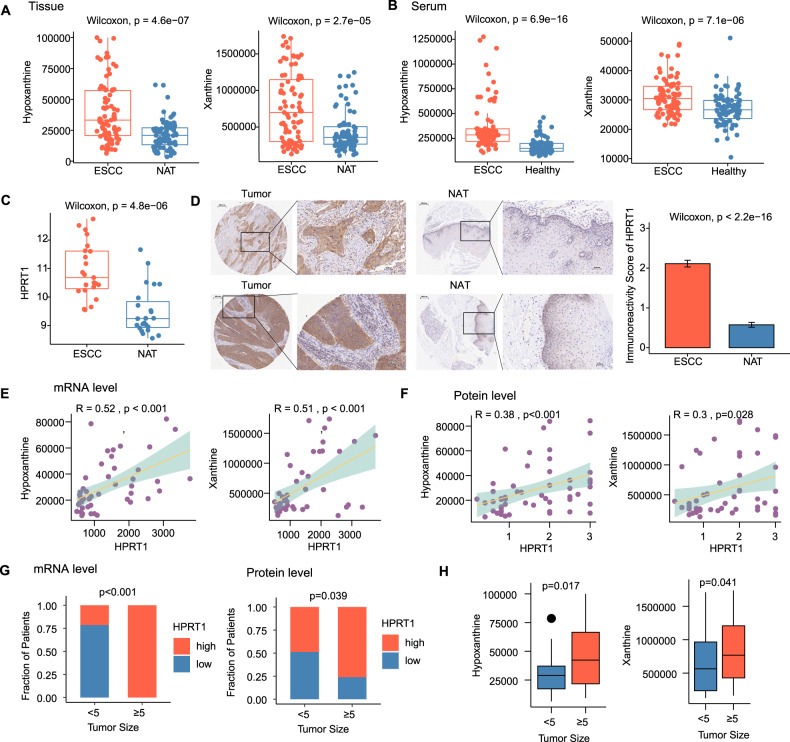
Network showing hypoxanthine/xanthine-HPRT1 is significantly upregulated and associated with malignant pathological characteristics in ESCC. **A** Boxplot showing significant differences in the levels of hypoxanthine/xanthine between ESCC tissues (*n* = 81) and NAT) (*n* = 81). Wilcoxon rank-sum test, *p* < 0.001. **B** Boxplot showing the significant difference in the levels of hypoxanthine/xanthine between the serum of patients with ESCC (*n* = 81) and serum of healthy subjects (*n* = 81).Wilcoxon rank-sum test, *p* < 0.001. **C** Boxplot showing the significant difference in HPRT1 mRNA levels between ESCC tissues (*n* = 23) and NAT (*n* = 23). Wilcoxon rank-sum test, *p* < 0.001. **D** Bar chart showing the IHC score of HPRT1 staining in ESCC tissues (*n* = 64) and NAT (*n* *n*= 64). Wilcoxon rank-sum test, *p* < 0.001. **E**, **F** Pearson correlation analysis of hypoxanthine/xanthine abundance and HPRT1 expression level in patients with ESCC. Correlation between HPRT1 mRNA expression level and hypoxanthine/xanthine abundance in 23 paired tissues (*n* = 46) of ESCC (**E**). Correlation between the immunoreactivity score of HPRT1 expression and hypoxanthine/xanthine abundance in 27 paired tissues (*n* = 54) of ESCC (**F**). **G** Fisher’s exact test showing the correlation between HPRT1 mRNA expression levels and ESCC tumor size (*n* = 23). According to the median expression level of HPRT1 mRNA in patients with ESCC, tumor tissues were grouped into high- and low-expression groups (left). Fisher’s exact test showing the correlation between HPRT1 protein levels and tumor size in patients with ESCC (*n* = 64). Tumor tissues were divided into high- and low-expression groups according to the median HPRT1 IHC score in patients with ESCC (right). **H** Wilcoxon rank-sum test showing the correlation between hypoxanthine/xanthine abundance and tumor size in patients with ESCC (*n* = 81). ESCC esophageal squamous cell carcinoma, NAT normal tissues adjacent to the tumor, IHC score immunohistochemical score.

Upregulation of the hypoxanthine and xanthine abundance in tissue samples (Fig. 3A) was consistent with that in the serum samples (Fig. 3B). In addition, tissue sequencing data suggested that HPRT1 mRNA expression was significantly enhanced in tumor tissues in comparison to that in NAT (*p* < 0.001; Fig. 3C). Immunohistochemical (IHC) staining of 64 paired tissue specimens from patients with ESCC (Supplementary Fig. 4A, B) suggested that the HPRT1 protein level was significantly elevated in tumor tissues compared with those in NAT (*p* < 0.001; Fig. 3D). Pearson correlation analysis revealed that the abundance of hypoxanthine and xanthine in tumor tissues was positively correlated with HPRT1 expression at both mRNA and protein levels (*p* < 0.05; Fig. 3E, F). Logistic regression analysis highlighted that upregulated expression of HPRT1 was significantly linked to a more advanced TNM stage, a higher degree of infiltration, the presence of regional lymph node metastasis, and increased tumor size (for patients with ESCC, the longest diameter of the tumor was considered the tumor size measurement; Supplementary Table 4). HPRT1 expression at both mRNA (*p* < 0.001) and protein (*p* = 0.039) levels were significantly upregulated in tumors of size ≥5 cm than in those of size <5 cm (Fig. 3G). The abundance of hypoxanthine (*p* = 0.017) and xanthine (*p* = 0.041) was significantly increased in tumors of size ≥5 cm than in those of size <5 cm (Fig. 3H). These results suggested that upregulated expression of the hypoxanthine/xanthine-HPRT1 network was associated with malignant pathological characteristics of ESCC.

To ascertain the level of hypoxanthine and xanthine catabolism in patients with ESCC, uric acid levels were analyzed through biochemical examination of blood samples of patients with ESCC. The process involved purine ring degradation with uric acid as the final product. The results indicate that the upregulation of purine salvage in ESCC did not significantly alter the abundance of uric acid, as illustrated in Supplementary Fig. 5.

### Result 4: Synergistic effect between hypoxanthine and HPRT1 contributes to the activation of the purine salvage pathway and promotion of ESCC development

**Fig. 4:**
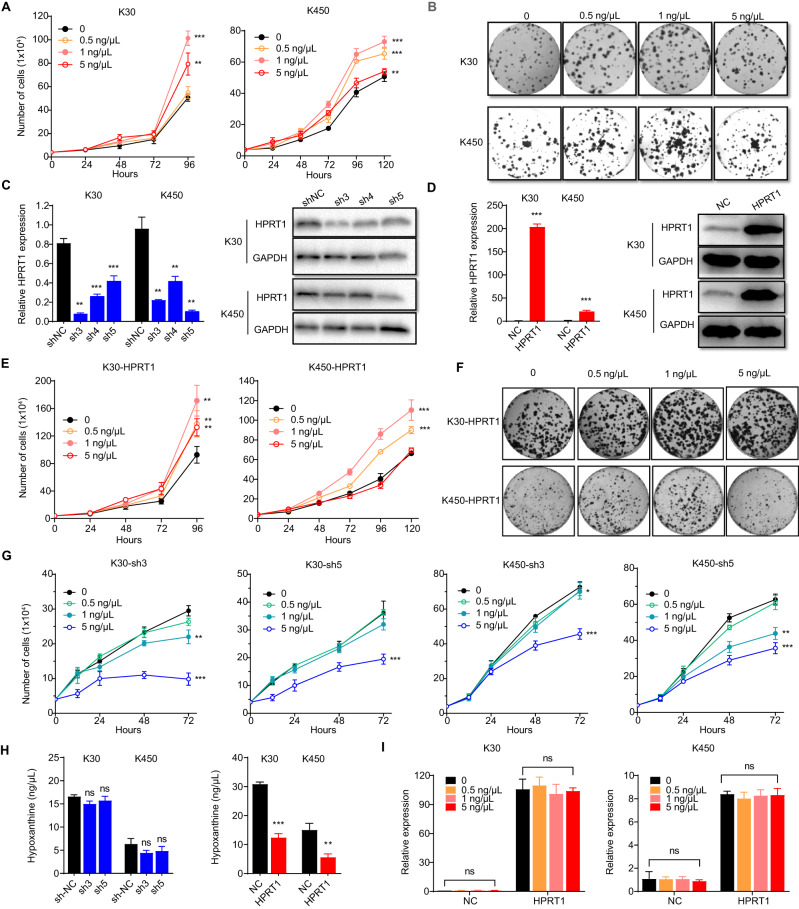
Synergistic action of hypoxanthine and HPRT1 in promoting ESCC development. **A** Hypoxanthine promoted the proliferation of KYSE-30 and KYSE-450 cells. Two-way ANOVA test, **p < 0.01, ***p < 0.001. **B** Hypoxanthine stimulated the colony formation of KYSE-30 and KYSE-450 cells. **C**, **D** Establishment of HPRT1 stably silenced and overexpressed cell lines verified at the mRNA and protein expression level in ESCC cells (KYSE-30 and KYSE-450).Two-tailed unpaired *t* test, **p < 0.01, ***p < 0.001. **E** HPRT1 overexpression potentiated the proliferation-promoting effect of hypoxanthine in KYSE-30 and KYSE-450 cells. Two-way ANOVA test, **p < 0.01, ***p < 0.001. **F** HPRT1 overexpression increased the clonogenicity effects of hypoxanthine in KYSE-30 and KYSE-450 cells. **G** HPRT1 silencing with shRNAs diminished the pro-proliferative effects of hypoxanthine in ESCC cells. Two-way ANOVA test, **p < 0.05, **p < 0.01, ***p < 0.001. **H** Hypoxanthine concentration in cells with HPRT1 overex*p*ression or silencing. Two-tailed unpaired *t* test, ns, not significant, *p < 0.05, **p < 0.01, ***p < 0.001. **I** Effect of hypoxanthine on the expression of the HPRT1 gene in KYSE-30 and KYSE-450 cells. Two-tailed unpaired *t* test, ns not significan. ESCC esophageal squamous cell carcinoma, shRNA short hairpin RNA.

The role of hypoxanthine was assessed to further explore purine metabolism dysregulation in ESCC progression. Hypoxanthine supplementation (1 ng/μL) significantly stimulated the proliferation of KYSE-30 or KYSE-450 cells (*p* < 0.001; Fig. 4A). Supporting this result, hypoxanthine supplementation (1 ng/μL) significantly enhanced the clonogenicity of ESCC cells (*p* < 0.05; Fig. 4B and Supplementary Fig. 6A). Thus, these results suggest that hypoxanthine supplementation strikingly promoted the viability of ESCC cells.

To elucidate the involvement of hypoxanthine-HPRT1 synchronization changes in ESCC, multiple ESCC cell lines were generated by lentiviral transduction to either stably overexpress or silence HPRT1. After selection by puromycin, HPRT1 expression significantly downregulated in KYSE-30 and KYSE-450 cells transduced by shRNAs (sh3, sh5 vs. shNC; *p* < 0.01; Fig. 4C). Notably, KYSE-30 and KYSE-450 cells transduced by full-length cDNA had significantly overexpressed HPRT1 (HPRT1 vs NC; *p* < 0.001; Fig. 4D). Cells with HPRT1 overexpression showed increased proliferation of KYSE-30 or KYSE-450 cells under hypoxanthine supplementation when compared with cells that were only under hypoxanthine supplementation (*p* < 0.001; Fig. 4E and Supplementary Fig. 6B, C). In line with this, HPRT1 overexpression significantly enhanced the colony-forming ability of ESCC cells that were under hypoxanthine supplementation (*p* < 0.05; Fig. 4F and Supplementary Fig. 6D). Notably, HPRT1 silencing–induced proliferation inhibition could not be rescued by hypoxanthine supplementation (Fig. 4G).

To gain more insight into the interaction between HPRT1 and hypoxanthine, hypoxanthine concentration in ESCC cells was examined using the ELISA kit. In comparison to control cells, a mild and insignificant reduction was observed in hypoxanthine concentrations in HPRT1-silenced cells (*p* > 0.05; Fig. 4H left). The data indicated significantly lower hypoxanthine levels in cells with HPRT1 overexpression (*p* < 0.01; Fig. 4H right). Furthermore, no significant difference was observed in HPRT1 mRNA expression in cells with silenced or overexpressed HPRT1 or in control cells after hypoxanthine treatment (*p* > 0.05; Fig. 4I and Supplementary Fig. 7). Collectively, these outcomes highlighted that higher HPRT1 expression levels in ESCC cells led to increased hypoxanthine utilization by the cells. Hypoxanthine-HPRT1 activity was simultaneously upregulated, thus possibly playing a synergistic role in promoting cell proliferation in ESCC.

### Result 5: HPRT1 promoted malignant proliferation of ESCC through phosphorylation of the YAP protein

HPRT1 has been shown to be involved in the pathogenesis of various human cancers, but its mechanism of action has not been reported in ESCC [24, 25]. Through the regulation of the purine salvage pathway, HPRT1 primarily participates in nucleotide synthesis [26]. Using data from the The Cancer Genome Atlas (TCGA) database, the HPRT1 expression profile and its potential for predicting prognosis was assessed in different cancer types. Significantly elevated HPRT1 expression levels were observed in 12 types of malignant tissues in comparison to the corresponding normal tissues (*p* < 0.05; Supplementary Fig. 8A and Supplementary Table 5). Next, Cox proportional risk model analysis was performed by acquiring survival data. The results suggested that, in 17 cancer types, the mortality risks were significantly elevated in the individuals in the group with high HPRT1 expression in comparison to those with low HPRT1 expression (Supplementary Fig. 8B and Supplementary Table 6). Remarkably, in esophageal cancer, the risk of mortality was significantly elevated in the group with high HPRT1 expression compared to the group with low expression (HRs = 2.215, 95% CI: 1.143–4.293, *p* = 0.018). The Kaplan-Meier survival curves highlighted that the OS of the HPRT1 high-expression group in esophageal cancer was shorter than that of the HPRT1 low-expression group (log-rank test, *p* = 0.016). Notably, the high HPRT1 expression group had a significantly shorter median OS of 18.2 months, compared to the low HPRT1 expression group with a median OS of 31.6 months (*p* = 0.018). The 3-year survival rates were 5% and 10.6%, respectively (Supplementary Fig. 8C). These data imply that HPRT1 may act as a novel oncogene in ESCC progression.

**Fig. 5:**
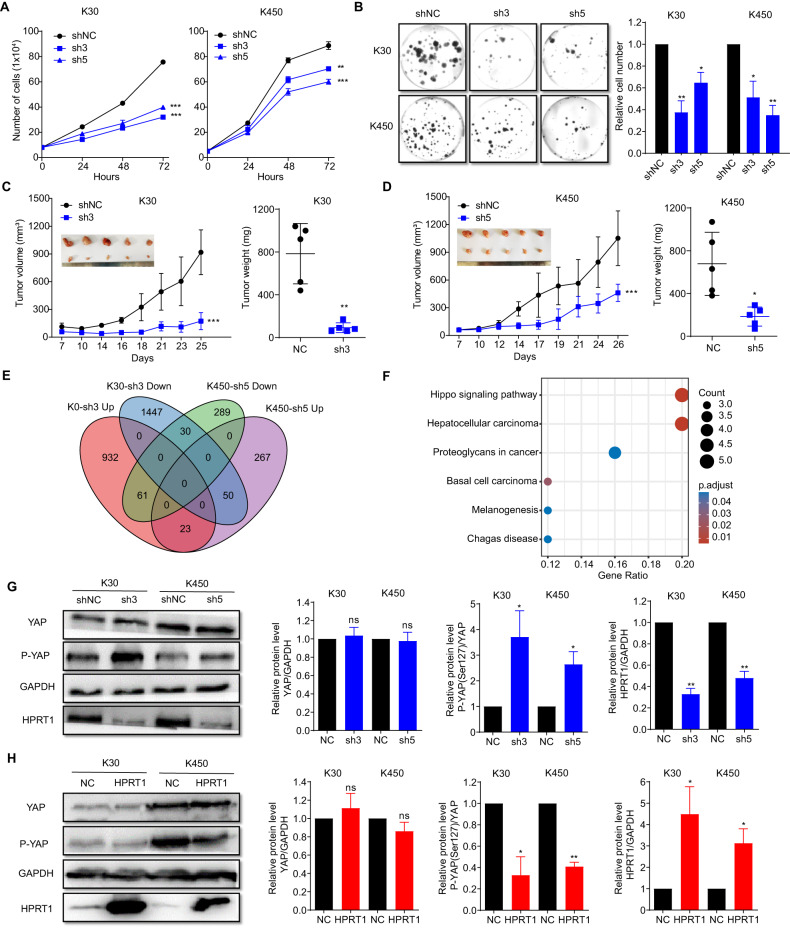
HPRT1 promoted the malignant proliferation of ESCC cells in vitro and in vivo. **A** HPRT1 silencing with shRNAs inhibited the proliferation of KYSE-30 and KYSE-450 cells. Two-way ANOVA test, **p < 0.01, ***p < 0.001. **B** Silencing of HPRT1 with shRNAs inhibited the clonogenicity of KYSE-30 and KYSE-450 cells. Two-tailed unpaired *t* test, *p < 0.05, **p < 0.01. **C**, **D** In vivo growth of ESCC xenografts was inhibited by HPRT1 silenced sh3 (KYSE-30) and sh5 (KYSE-450) vs corresponding shNC. **E** Venn diagram showing HPRT1 silencing affects gene expression in KYSE-30 and KYSE-450 cells. **F** KEGG enrichment pathways of the DEGs in both HPRT1-silenced KYSE-30 and KYSE-450 cells. **G**, **H** HPRT1 suppressed the phosphorylation of the YAP protein in KYSE-30 and KYSE-450 cells. Two-tailed unpaired *t* test, ns, not significant, *p < 0.05, **p < 0.01. ESCC esophageal squamous cell carcinoma, shRNA short hairpin RNA, DEGs differentially expressed genes, KEGG Kyoto Encyclopedia of Genes and Genomes.

The biological function of HPRT1 in ESCC was further explored both in vitro and in vivo. The silencing of HPRT1 resulted in significant inhibition of the proliferation of KYSE-30 and KYSE-450 cells (*p* < 0.01; Fig. 5A), whereas its overexpression significantly increased the proliferation of ESCC cells (*p* < 0.01; Supplementary Fig. 9A). Consistent with these results, silencing of HPRT1 inhibited clone formation in KYSE-30 and KYSE-450 cells (*p* < 0.05, Fig. 5B), and its overexpression enhanced the clonogenicity of cells (*p* < 0.05, Supplementary Fig. 9B). These findings suggest that HPRT1 is a novel oncogene in ESCC pathogenesis. Furthermore, HPRT1 exhibited a significant promotion of migration in ESCC cells (Supplementary Fig. 9C–E). The in vivo effects of HPRT1 were assessed by xenografting ESCC in nude mice. The data acquired indicated that these xenografts with stably silenced HPRT1 showed delayed growth and had significantly reduced tumor volume and mass in comparison to the control mice (*p* < 0.05; Fig. 5C, D). These outcomes indicated that HPRT1 silencing can inhibit the in vivo proliferation of malignant ESCC cells.

To elucidate the involvement of HPRT1 in regulating crucial tumorigenic genes and downstream pathways, RNA-seq analysis was conducted on KYSE-30 and KYSE-450 cells with stable silencing of HPRT1, followed by comparing them with control counterparts. Differential gene expression analysis was conducted on the RNA sequencing data, revealing that these cell lines depicted concurrent alterations in the expression of 53 genes. Among these genes, 30 exhibited downregulated expression, while 23 exhibited upregulated expression, as depicted in Fig. 5E. Next, pathway enrichment analysis was conducted on the co-altered differential genes (Fig. 5F). The results indicated that the Hippo signaling pathway was highly affected in both ESCC cell lines owing to HPRT1 silencing (Fig. 5F). Furthermore, this HPRT1 silencing significantly promoted the phosphorylation of the YAP protein at Ser127 compared with that in control cells (*p* < 0.01), with no significant difference observed in the protein expression (*p* > 0.05, Fig. 5G and Original western blots). Similarly, HPRT1 overexpression downregulated YAP phosphorylation levels in ESCC cells (*p* < 0.01), with no significant difference in YAP protein expression observed (*p* > 0.05, Fig. 5H and Original western blots). Collectively, these data imply that HPRT1 may promote cancer progression by inhibiting YAP protein phosphorylation in the downstream Hippo signaling pathway.

## Discussion

This study comprehensively evaluated the metabolic dysregulation of ESCC through an integrated strategy of metabolomics and transcriptomics. A group of purine salvage–associated metabolites were identified, and the expression of these metabolites was dysregulated in the blood in a tissue-specific manner. Furthermore, a diagnostic model was developed using two purine salvage–associated metabolites. This model could accurately discriminate patients with ESCC from normal individuals. The diagnostic sensitivity of this model was found to be better than that of CEA, CYFRA21-1, and SCC [15, 27–30], which are the biomarkers currently used in clinical practice. This report presented two noteworthy findings. The first one is the identification of hypoxanthine/xanthine as a promising candidate biomarker for ESCC diagnosis. The second is the synergistic effect between hypoxanthine and HPRT1 in terms of promoting ESCC progression. Collectively, these outcomes not only identified key metabolites associated with ESCC but also enhanced our comprehension of the underlying mechanisms of ESCC metabolism.

In our previous study, 653 serum samples were collected, representing different stages ranging from normal to ESCC progression. These samples consisted of 305 normal cases, 77 cases of esophagitis, 228 cases of low-grade dysplasia, and 43 cases of high-grade dysplasia/ESCC. The ordinal information of these four groups was imported into a statistical analysis to investigate serum metabolites associated with the progression of ESCC. The metabolites hypoxanthine, inosine, carnitine (14:1), glycochenodeoxycholate, and PC (P-18:0/18:3) are potential biomarkers for risk prediction and early diagnosis of ESCC [23]. However, the biological information provided by metabolomics remains limited as it unravels only a one-dimensional perspective to understand metabolic dysregulation in tumors [31, 32], which may result in false-positive biomarkers. The development of effective diagnostic approaches requires a system-level comprehensive analysis of the molecules altered in a specific disease [33, 34]. Therefore, the integrative analysis of metabolomics with other omics data is a crucial step in identifying biomarkers and the disease etiology [35, 36]. Genes and their corresponding proteins play important roles in the production and regulation of metabolites [37]. Transcriptomics can help examine all RNAs expressed by genes at a specific time point, in a certain environment and is an essential means to reveal the mechanism of a particular disease [38, 39]. In this research, metabolomics and transcriptomics were integrated to analyze metabolic alterations in ESCC [40], further verifying from two dimensions that hypoxanthine and xanthine are crucial biomarkers of ESCC.

Although emerging evidence suggests the potential of metabolic biomarkers in tumor diagnosis [41, 42], how the relevant metabolites are involved in ESCC development requires a systematic investigation. In this study, it was shown that hypoxanthine/xanthine-HPRT1 is one of the most significantly upregulated gene-metabolite networks in ESCC. Its high expression levels were correlated with the pathological characteristics of patients with ESCC. Rapidly proliferating tumor cells have a high demand for nucleotides due to the need for ample templates to replicate the DNA genome during cell division and for the synthesis of rRNA and mRNA for protein production [43]. In most cells, purine nucleotides can be synthesized through two pathways: the salvage pathway and the de novo pathway. These pathways sequentially construct purine nucleotides starting from phosphoribosyl pyrophosphate (PRPP) [44, 45]. The HPRT1 gene, located on the X chromosome, encodes the HGPRT enzyme, which contributes to recycling nucleotides for use in DNA and RNA synthesis in actively dividing cells The purine salvage pathway begins with the PRPP-dependent ribosylation of hypoxanthine by HGPRT, resulting in the formation of inosine monophosphate (IMP). IMP can be further utilized in the guanylate metabolite pathway for guanosine triphosphate (GTP) biosynthesis or undergo amination to eventually form adenosine triphosphate (ATP) [46]. Similarly, xanthine can be salvaged for GTP production or utilized for ATP production. The results suggest that purine salvage synthesis, not de novo synthesis, may serve as the fundamental mechanism to replenish purine pools in ESCC [47]. Hypoxanthine aids in maintaining a balance between the adenylate and guanylate pools within the cell [48]. Hypoxanthine/xanthine is taken up and released by equilibrative nucleoside transporter 2 [49, 50], which allows them to be detected in the blood.

Furthermore, it was demonstrated that hypoxanthine synergizes with oncogene HPRT1, contributing to promoting ESCC development. Moreover, upregulated HPRT1 expression was detected in tumor specimens and was associated with evidently shortened survival time in patients with ESCC. Consistently, HPRT1 exhibited strong oncogenic potential both ex vivo and in vivo. Specifically, in ESCC, HPRT1 has the potential to inhibit the phosphorylation of the YAP protein at Ser127. This inhibition leads to an increased formation of nonphosphorylated YAP, which can then translocate into the nucleus and facilitate transcriptional activity. Consequently, this mechanism promotes cell proliferation [51]. According to the present literature, this is the first study to report a network demonstrating the dysregulation of gene-metabolite in ESCC, wherein the upregulated network of hypoxanthine/xanthine-HPRT1 is likely to play a crucial role in the progression of ESCC.

However, there are certain limitations to this research that need to be acknowledged. Firstly, it is crucial to collect a larger number of samples, particularly blood samples from patients with early-stage ESCC, to validate the potential significance of purine salvage-related metabolites (hypoxanthine/xanthine) in the detection and screening of early-stage ESCC. Additionally, even though the discovery cohort included a significant proportion (53.09%) of patients with early-stage ESCC based on pathological diagnosis, further studies with a larger and more diverse cohort are needed to enhance the external validity and generalizability of the identified biomarkers. However, the findings of this report do not establish a causal correlation, despite its emphasis on biologically plausible mechanisms. To enhance the robustness and reliability of the results, metabolomics and transcriptomics data were integrated to reveal dysregulated gene-metabolite networks in ESCC. Furthermore, preliminary biological experiments were conducted to elucidate the mechanism underlying the synergistic promotion of ESCC progression by hypoxanthine-HPRT1. In the future, a deeper understanding of the metabolic adaptation mechanisms, particularly in relation to the rewriting principles of the purine salvage pathway, may ultimately contribute to improved strategies for preventing the onset and progression of ESCC.

## Conclusions

In this study, a comprehensive strategy was utilized to identify a dysregulated metabolic network associated with ESCC progression, involving the metabolite biomarkers hypoxanthine and xanthine, the HPRT1 gene, and the purine salvage pathway. The findings of this research provide valuable support for the establishment of novel diagnostic strategies for early ESCC and partly enhance our understanding of the molecular mechanisms involved in ESCC progression.

## Materials and methods

### Study cohorts and sample collection

Prior to conducting the study, approval was obtained from the Ethical Committee of Shandong Cancer Hospital and Institute. Written informed consent was obtained from all participants involved in the study. The staging of all cases followed the guidelines provided by the American Joint Committee on Cancer/Union for International Cancer Control for cancer staging (8th edition, 2017). For the discovery cohort, 81 patients with ESCC and 81 healthy volunteers were recruited from Shandong Cancer Hospital and Institute (Shandong, China) and the three Upper Gastrointestinal Cancer Screening Bases of Shandong Province (Feicheng, Dongping, and Ningyang, Shandong, China) between October 2018 and March 2020. Paired tissue samples and serum samples were collected from patients with ESCC, and serum samples were collected from healthy volunteers. Furthermore, the population in the external validation cohort was enrolled from Shandong Cancer Hospital and Institute and two Upper Gastrointestinal Cancer Screening Bases (Wuwei, Gansu, China; Feicheng, Shandong, China) between August 2019 and October 2020. The validation cohort comprised 220 participants classified into four populations: healthy volunteers (*n* = 58), patients with ESCC (*n* = 81), lung cancer (*n* = 41), and colorectal cancer (*n* = 40). Serum samples were collected from all participants. Further details about participant eligibility criteria and sample collection are provided in the *Supplementary methods*.

### Metabolic profiling of ESCC

For metabolite profiling, an ultra-high-performance liquid chromatography (UHPLC) system (1290 series, Agilent Technologies, USA) was utilized in conjunction with a quadruple time-of-flight mass spectrometer (TripleTOF 6600; AB SCIEX, USA). Metabolomics analysis of samples was performed as previously described with slight modification [52]. Further details are provided in the *Supplementary Methods*.

### RNA sequencing of tissue samples

RNA sequencing and differential gene analysis of ESCC tissues were conducted at Berry Oncology Corporation (Beijing, China). A total of 3110 mRNAs were identified in the ESCC tissues. Further details are provided in the *Supplementary Methods*.

### Immunohistochemistry assay

Two microarrays of human ESCC tissues were used for this assay. One microarray included tumor tissue specimens (*n* = 35) and NAT specimens (*n* = 35), collected by our research group, designed and produced by Shanghai Outdo Biotech Co.LTD. Another microarray included 29 pairs of ESCC tissues purchased from the same company, with detailed pathological data. HPRT1 (ab109021; Abcam) antibody was used at a 1:4000 dilution. Further details are provided in the *Supplementary Methods*.

### Quantification of hypoxanthine/xanthine detection by ELISA

Hypoxanthine/xanthine can be oxidized by a xanthine enzyme mixture to form a specific intermediate, which then reacts with the chromogen and probe to form the final product, detected by the colorimetric method (λ = 570 nm). Hypoxanthine/xanthine levels in the serum and cell lines of the validation cohort were quantified using the Hypoxanthine/Xanthine Assay Kit (ab155900; Abcam).

### Pan-cancer analysis based on the TCGA database

RNA expression data were acquired from TCGA database (https://xenabrowser.net/datapages/), including information on all cancer types, with the data of both cancer and corresponding normal tissues extracted to compare differences in HPRT1 expression (Supplementary Table 5). Survival data were retrieved in all cancer types to compare the prognostic differences among patients with different HPRT1 expression levels (Supplementary Table 6).

### Cell culture

The human ESCC cell line KYSE-450 was obtained from the China Center for Type Culture Collection. The human ESCC cell line KYSE-30 was obtained from the Key Laboratory of Shandong Cancer Hospital and Institute. Further details are provided in the *Supplementary Methods*.

### Lentiviral transduction

The lentiviral GV112-hU6-MCS-CMV-Puro-sh3/sh4/sh5 and control (shNC) vectors were provided by the Shanghai GeneChem Corporation (China). The lentiviral LV18-CMV-Puro-HPRT1 and control (NC) vectors were purchased from Shanghai GenePharma Corporation (China). Further details are provided in the *Supplementary Methods*.

### Quantitative reverse transcription–polymerase chain reaction

Total RNA was extracted from the cultured cells using the RNAsimple Total RNA Kit (TIANGEN, Beijing, China). For complementary DNA (cDNA) synthesis, each RNA sample was reverse transcribed using PrimeScript™ RT Master Mix (RR036A; TaKaRa). Relative mRNA levels were quantified according to the 2^−ΔΔCt^ method. The primers used for this assay are listed in Supplementary Table 7.

### Western blot assay

Western blot assay was conducted following the standard protocol. Antibodies against the YAP protein (Phospho-YAP/TAZ Antibody Sampler Kit, 52420, Cell Signaling Technology), Phospho-YAP (Phospho-YAP/TAZ Antibody Sampler Kit, 52420, Cell Signaling Technology), HPRT1 (ab109021; Abcam), and GAPDH (ab8245; Abcam) were utilized. Protein visualization was performed using the Electrochemiluminescence Western Blotting Substrate (WBKLS0100; Millipore).

### Cell proliferation assays and hypoxanthine effect analyses

To perform cell proliferation assays, stably transfected KYSE-30 (4 × 10^4^ cells per well) or KYSE-450 (8 × 10^4^ cells per well) cells were seeded in 12-well plates. For the colony formation assays involving hypoxanthine effect analysis, 4 × 10^4^ stably transfected KYSE-30 and KYSE-450 cells were seeded per well in 12-well plates. Hypoxanthine, diluted in phosphate-buffered saline (PBS), was added to each well to achieve the desired final concentrations (0, 0.5, 1, and 5 ng/μL). The ESCC cells were harvested and counted at 24, 48, 72, 96, and 120 h after seeding.

### Colony formation assays and hypoxanthine effect analyses

Stably transfected KYSE-30 cells (800 cells per well) were seeded in a 6-well cell culture plate. After 10 days, when colonies became visible, the cells were washed twice with cold PBS and fixed with methanol. Following fixation, the cells were stained with 1% crystal violet, and the number of ESCC colonies formed in each well was counted. Similarly, stably transfected KYSE-450 cells (1000 cells per well) were seeded in a 6-well cell culture plate, and the number of KYSE-450 cell colonies was counted after 14 days. For colony formation assays investigating the effect of hypoxanthine, stably transfected KYSE-30 and KYSE-450 cells (800 cells per well) were seeded in a 6-well cell culture plate. Hypoxanthine concentrations were used as described above.

### Transwell assays

Transwell chambers were prepared by coating them with 60 μL of Matrigel (diluted 1:20; pore 8 μm, Corning) and incubating them for 4 h in a 5% CO_2_ incubator. In the upper chamber of the Transwell setup, KYSE-30 cells or KYSE-450 cells were added, along with a culture medium supplemented with 1% FBS. The lower chamber of the Transwell setup was added with 650 μL of medium supplemented with 10% FBS. After 48 h, the cells that had migrated to the lower chamber through the pores were stained with a 0.1% crystal violet solution and then counted.

### Wound healing assays

KYSE-30 and KYSE-450 cells were cultured until they reached approximately 90% confluence. A wound was created by scratching the cell layer using a 200-μL pipette tip. The ESCC cells were then incubated at 37 °C with 5% CO_2_. The extent of wound closure was measured and quantified by assessing the average closure of the wound area.

### Mouse xenograft tumor models

To evaluate the in vivo role of HPRT1, female nude BALB/c mice (4–6 weeks old; obtained from Beijing Vital River Laboratory Animal Technology Co. Ltd., Beijing, China) were subcutaneously inoculated with HPRT1-silenced or control (shNC) KYSE-30 (5 × 10^6^ cells) or KYSE-450 (8 × 10^6^ cells) cells into the fossa axillaries (*n* = 5 per group). Mice were randomized to each experimental group. Tumor growth was monitored every 2 days after the tumor volume reached 100 mm^3^. All animal studies were conducted following the guidelines and regulations approved by the Animal Care and Use Committee of Shandong Cancer Hospital and Institute.

### Statistical analysis

R (version 3.5.0) was used for the analysis of omics data. An unsupervised PCA was conducted to visualize the global metabolic profiles among groups, utilizing the R function “prcomp”. Subsequently, a supervised model of PLS-DA was employed to assess the global metabolic difference between groups. To assess the validity of the discriminant models and prevent overfitting, a permutation test was performed 200 times. Additionally, the VIP value was calculated for each variable in the PLS-DA model. Differential analysis between sample groups was performed using the Wilcoxon rank-sum test and Benjamin-Hochberg FDR adjustment. Dysregulated metabolites meeting the criteria of FDR-adjusted *p* < 0.05 and VIP > 1 were selected. For analysis, the R package function “randomForest” implemented a random forest regression model. ROC curves were constructed using the R package function “pROC” to evaluate the diagnostic performance of the metabolite biomarkers. The AUC and a 95% CI were calculated to assess predictive accuracy.

The R package function “edgeR” was utilized to analyze the differential expression of genes between groups, obtaining the adjusted *p* value (p_adj_) based on FDR. DEGs were identified based on the criteria of *p*_adj_ < 0.05 and |log2(FoldChange)| > 1. Pathway enrichment analysis involved tests for hypergeometric distribution, and the Kyoto Encyclopedia of Genes and Genomes database (KEGG, http://www.genome.jp/kegg/) was used for mapping the differential metabolites and DEGs. The Wilcoxon rank-sum test was conducted to compare differences in HPRT1 expression between groups. Fisher’s exact test was used to compare HPRT1 expression with pathological characteristics of patients with ESCC, whereas their correlation was assessed through logistic regression analysis. Pearson correlation analysis was conducted to examine the correlation between metabolite levels and HPRT1 expression. Concerning the TCGA data, the Wilcoxon rank-sum test was performed to compare HPRT1 expression across different cancer types, and survival analysis was performed to evaluate the prognosis of patients with different types of cancer. The R package “surminer” was utilized to determine the optimal threshold of HPRT1 expression, and Kaplan-Meier survival curves were constructed using the R package “survival” to assess survival differences among patients with different HPRT1 expression levels. Transcriptome sequencing of ESCC cells following HPRT1 silencing was performed, and DEGs were subjected to functional enrichment analysis using the R package function “clusterProfiler.”

The data of the biological experiments were statistically analyzed using GraphPad Prism 9. For statistical comparisons between two groups, we used the two-tailed unpaired *t* test. For comparisons between more than two groups or conditions, we applied the two-way ANOVA test. All experiments were repeated at least three times. The tests utilized are two-tailed, and adjustments are made for multiple comparisons. The data are presented as the mean ± standard deviation. A *p* value of less than 0.05 was considered to indicate statistical significance.

### Supplementary information

Supplementary materials

checklist

Original western blots

## Data Availability

The raw sequence data reported in this paper have been deposited in the Genome Sequence Archive (Genomics, Proteomics & Bioinformatics 2021) in National Genomics Data Center (Nucleic Acids Res 2022), China National Center for Bioinformation/Beijing Institute of Genomics, Chinese Academy of Sciences (GSA-Human: HRA005109, HRA005172) that are publicly accessible at https://ngdc.cncb.ac.cn/gsa-human.
