# Supplementary material for: Purine salvage–associated metabolites as biomarkers for early diagnosis of esophageal squamous cell carcinoma: a diagnostic model–based study
Source: Cell Death Discov. 2024 Mar 14;10:139. doi: 10.1038/s41420-024-01896-6 (PMC10940714; doi:10.1038/s41420-024-01896-6)
Supplement: Supplementary file 1 — Supplementary materials [file 41420_2024_1896_MOESM1_ESM.pdf]

Supplemental materials

Supplemental methods

Participant eligibility criteria

The eligibility criteria for participants in discovery cohort. ESCC patients: (1) not receiving any anti-tumor therapy; (2) no history of other malignant tumors; (3) not suffering from metabolic diseases, liver diseases or kidney diseases; (4) underwent ESCC surgery, and had been diagnosed and confirmed by two pathologists via histopathologic examinations. Healthy volunteers: (1) underwent endoscopy screening without any upper gastrointestinal lesions; (2) no history of malignant tumors; (3) not suffering from metabolic diseases, liver diseases or kidney diseases; (4) had normal results of physical examination and laboratory test.

The eligibility criteria for participants of validation cohort were set as follows: patients with clinically diagnosed esophageal, lung and colorectal cancers according to the AJCC/UICC staging criteria (8th edition, 2017) for corresponding cancer.

Sample collection

Blood sample collection. Vacuum blood collection tube (yellow, 5 mL) with separation gel and coagulant was used for collecting overnight fasting blood sample for each participant. The fresh blood was centrifuged at 3,000 rpm for 10 min, and then supernatant (400 μL per tube) was extracted and immediately frozen at -80℃ refrigerator until metabolomic analysis.

Tissue sample collection. Tissue samples were taken from surgical specimens of ESCC patients during the surgery. Tumor tissues and the corresponding normal tissues

adjacent to the tumor (NAT, at least 2 cm away from the outer edge of the suspicious lesion) were sampled. Samples for metabolomic analysis and sequencing were frozen in liquid nitrogen within 30 min of extraction from the body and stored at -80°C. Another set of matched tissue samples were soaked in formalin and then fixed in paraffin for immunohistochemical staining. Samples were annotated with full clinical details including demographic data, comorbidities, drug intakes, etc.

Reagents and materials.

Liquid chromatography-mass spectrometry (LC-MS) grade water (H2O), acetonitrile (ACN), methanol (MeOH), 0.1% formic acid (FA) in water and 0.1% FA in ACN were purchased from Honeywell (Muskegon, MI, USA). Ammonium hydroxide (NH4OH) and ammonium acetate (NH4OAc) were purchased from Sigma-Aldrich (St. Louis, USA) and dissolved in LC-MS grade water prior to use.

Metabolite extraction

All samples were blindly labeled by serial sample collection numbers. The orders of both serum and tissue samples were randomized by blocks prior to metabolite extraction and LC-MS analysis. After thawed at 4°C on ice, a volume of 50 μL of each serum sample was pipetted into a 96-well plate, then extracted with 150 μL of MeOH (kept at -20 °C before extraction) using the Bravo liquid handling system (Agilent Technologies, USA). Subsequently, the plate was vortex for 30 s and incubated for 2 h at -20°C to precipitate proteins. The 96-well plate was then centrifuged at 4,000 rpm for 20 min at 4°C, and the supernatants were transferred into LC-MS vials and stored at -80°C until the LC-MS analysis. A Quality control (QC)

sample was prepared by pooling a small aliquot from each serum sample (including all of serum samples from healthy volunteers and ESCC patients), and extracted together with other serum samples.

Tissue samples (10.00 ± 1.00 mg for each sample) were first homogenized with ceramic beads in 200 μL of H2O for three times using a Precellys homogenizer (6,000 rpm). The homogenization took 20s with 5s intervals each time, and liquid nitrogen was used to keep the low-temperature of homogenization. After homogenization, a volume of 800 μL of MeOH:ACN (1:1, v/v) was added as extraction solvent, then vortex for 30 s, and incubated in liquid nitrogen for 1 min. The samples were subsequently thawed at room temperature, and sonicated for 10 min at 4°C. This vortex-freeze-thaw cycle was repeated three times. To precipitate proteins, the tissue samples were incubated at -20°C for 1 h, followed by a centrifugation at 13,000 rpm for 15 min at 4°C. Then the supernatant was taken and evaporated to dryness in a vacuum concentrator. Dry extracts were then reconstituted with a 100 μL of ACN:H2O (1:1, v/v), followed by sonicated for 10 min. Then, it was centrifuged at 13,000 rpm for 15 min at 4°C to remove insoluble debris. Finally, the supernatant was also transferred to LC-MS vials and stored at -80°C prior to the LC-MS analysis. A QC sample was prepared by pooling a small aliquot from each homogenized tissue solution sample (including all of the tumor tissue samples and adjacent controls), and extracted together with other tissue samples.

Metabolic profiling of ESCC

A Waters BEH Amide column (1.7 μm; 2.1 × 100 mm) was used for the LC separation,

and the column temperature was maintained at 25°C. The flow rate was set as 0.5 mL/min, and the sample injection volume was 2 μL. The mobile phase A was 25 mM ammonium hydroxide (NH4OH) + 25 mM ammonium acetate (NH4OAc) in water, and B was ACN in both positive mode (ESI+) and negative mode (ESI-). The column was eluted with a linear gradient system (B %): 0 min, 95%; 0.5 min, 95%; 7 min, 65%; 8 min, 40%; 9 min, 40%; 9.1min, 95%; 12min 95%. The autosampler was set at 4°C. The TOF mass range was set as m/z 50-1200 Da in both positive mode (ESI+) and negative mode (ESI-). The source parameters were set as follows: GAS1, 60 psi; GAS2, 60 psi; CUR, 30 psi; TEM, 600°C; and ISVF: 5000 V and -4500 V in positive and negative modes, respectively. QC samples were analyzed every eight injections of biological samples and a blank sample (ACN : H2O, 1:1, v/v) to monitor the stability of the data acquisition and used for data normalization.

Metabolomic data processing

First, ProteoWizard (version 3.06150) was used to convert raw MS data (.d) files to the mzXML format, and R package "XCMS" (version 3.2) was used for data processing. The generated data matrix consisted of the mass-to-charge ratio (m/z) value, retention time (RT), and peak abundance. R package "CAMERA" was used for peak annotation after XCMS data processing. Metabolic peaks detected less than 50% in all the QC samples were excluded. Subsequently, the R package "MetNormalizer" was used for the normalization of each metabolic peak in subject samples to remove unwanted system error that occurred among intra- and interbatches. Minimum value (half of the least nonzero value) or a random forest (RF) regression model (R

packages “missForest”) was used for missing data imputation before differential analysis and correlation analysis, respectively. The combination of accurate mass and experimental MS/MS match against our in-house tandem MS spectral library and other databases (NIST, METLIN, and MassBank) is used for metabolite identification. Moreover, we applied the Metabolite annotation and Dysregulated Network Analysis (MetDNA) strategy developed earlier by our research group.

RNA sequencing for tissue samples

Briefly, ribosomal RNA was first removed by Epicentre Ribo-zero rRNA Removal Kit (Epicentre, USA), and rRNA free residue was cleaned up by ethanol precipitation. Subsequently, sequencing libraries were generated by RNA Library Prep Kit (NEB, USA) for Illumina using the rRNA-depleted RNA. Next, products were purified (AMPure XP system, Beckman Coulter, Beverly, USA) and library quality was assessed on the Agilent Bioanalyzer 2100 system. The clustering of the index-coded samples was performed on a cBot Cluster Generation System using TruSeq PE Cluster Kit v3-cBot-HS (Illumia, USA). After cluster generation, the libraries were sequenced on an Illumina Hiseq 4000 platform and 150 bp paired-end reads were generated.

Immunohistochemical staining

Scoring. The final staining index was calculated using the formula: positive-staining score × staining intensity score. The staining intensity score: Under a low magnification microscope, the pathologist observed the entire tissue point, and classified the tissue point as weak intensity, moderate intensity, and strong intensity.

Weak intensity was scored as 1; moderate intensity was scored as 2; strong intensity was scored as 3. The positive-staining score: First, the pathologist observed and selected three fields of view with different staining intensities under a low-power microscope for one tissue point, and then scored (0% - 100%) the fields under a high-power microscope. The HPRT1 protein was localized in the cytoplasm. Therefore, the pathologist evaluated the three visual fields separately, and took the average value as the positive-staining score for the tissue point.

Cell Culture and Cell Transfection.

The cells were maintained in DMEM medium (Gibco, C11995500BT) supplemented with 10% fetal bovine serum (FBS; BI, 04-001-1) and 1% penicillin–streptomycin antibiotics (Gibco, 15140-122) at 37°C in a 5% CO^2 incubator. The cells were periodically tested and found to be negative for mycoplasma contamination.

Cells in an exponential growth phase were subcultured in 6-well plates to ensure 40% - 50% cell confluence by the 24 h for transfection. After 48 h of transduction, the cells were selected with puromycin (Gibco, USA) for one week, and the surviving cells were continuously cultured for subsequent experiments.

Supplemental tables

Supplemental table 1. Information of 36 metabolites in serum discovery cohort.

NO.	ESI[a]	m/z[b] (Da)	RT[c] (second)	Metabolites	FDR[d]	VIP[e]	Fold change
1	NEG	102.05524	388.863	(S)-2-Aminobutanoate	1.19E-10	1.48	1.51
2	POS	118.08647	267.4	Betaine	7.38E-05	1.06	0.87
3	POS	118.08587	295.5905	Dopamine	1.18E-07	1.36	0.77
4	NEG	119.03388	297.189	D-Erythrulose	6.01E-13	1.49	0.83
5	NEG	132.02936	395.833	Iminoglycine	3.95E-08	1.32	1.34
6	POS	137.04536	166.072	Hypoxanthine	6.21E-15	1.45	2.15
7	NEG	146.08114	290.942	4-Aminobutyraldehyde	1.72E-10	1.55	0.79
8	NEG	146.04509	388.798	L-Glutamate	5.06E-10	1.46	1.43
9	POS	148.05967	385.051	4-Aminocatechol	1.62E-12	1.49	1.42
10	NEG	151.02535	211.373	Xanthine	2.27E-05	1.08	1.16
11	NEG	173.05571	312.078	L-Aspartate	2.09E-05	1.13	0.83
12	NEG	177.03952	147.697	L-Galactono-1,4-lactone	1.57E-05	1.07	0.81
13	NEG	179.05608	296.396	Glycerone	1.91E-12	1.41	0.83
14	NEG	181.04944	187.793	3-(4-Hydroxyphenyl)lactate	2.21E-06	1.19	0.78
15	NEG	188.93833	317.0975	3-Sulfopyruvate	7.47E-09	1.41	0.85
16	NEG	190.05329	195.101	N-Benzyloxycarbonylglycine	7.43E-15	1.76	1.63
17	POS	203.05185	322.483	3-Deoxy-D-manno-octulosonate	6.52E-15	1.79	0.8
18	NEG	205.08242	329.564	(R)-3-Ureidoisobutyrate	2.55E-06	1.17	1.39
19	NEG	221.01104	223.361	cis-4,5-Dihydroxycyclohexa-1(6),2-diene-1,2-dicarboxylate	5.27E-12	1.63	1.51
20	NEG	221.09168	253.908	N5-Phenyl-L-glutamine	4.24E-09	1.37	1.45
21	NEG	222.13361	259.293	N8-Acetylspermidine	7.67E-05	1.02	1.19
22	NEG	227.12766	291.4655	Carboxynorspermidine	8.19E-05	1.02	0.62
23	POS	232.13992	430.088	N(omega)-Hydroxyarginine	2.81E-16	2.05	2.75
24	NEG	233.01991	273.079	Dehydroascorbate	2.48E-07	1.2	0.86
25	NEG	240.03481	196.212	Indolelactate	5.27E-12	1.67	0.67
26	POS	248.12364	337.942	6-Acetamido-2-oxohexanoate	3.06E-09	1.5	0.68
27	POS	259.09168	437.374	2-Amino-2-deoxy-D-gluconate	4.34E-04	1.03	1.43
28	POS	269.12526	193.503	Nopaline	1.51E-08	1.32	0.44
29	NEG	275.08705	447.646	(5-L-Glutamyl)-L-glutamate	1.35E-13	1.39	2.76
30	NEG	286.09255	123.067	Thiamine	3.62E-18	1.92	10.68
31	NEG	287.13432	329.818	Deoxyguanidinoproclavaminic acid	4.25E-08	1.34	1.73
32	NEG	300.03828	208.9685	Isoniazid alpha-ketoglutaric acid	1.92E-04	1.02	1.18
33	NEG	308.09682	366.72	N-Acetylneuraminate	3.10E-13	1.16	1.63
34	NEG	355.08609	101.974	Portulacaxanthin II	1.04E-12	1.69	0.7
35	POS	70.0653	305.578	Diethanolamine	7.16E-10	1.33	0.83
36	POS	84.044	216.246	1-Aminocyclopropane-1-carboxylate	7.31E-07	1.18	0.82

[a]ESI: electrospray ionizaiton mode, POS represents positive ion mode, NEG represents negative ion mode; [b]m/z: mass-to-charge ratio; [c]RT: retention time; [d]FDR: false discovery rate; [e]VIP: variable important in the projection.

Supplemental table 2. Dysregulated pathways in ESCC.

[a]KEGG	Pathway Name	Pathway length	Overlap	*p* value
hsa04080	Neuroactive ligand-receptor interaction	393	84	<0.001
hsa04024	cAMP signaling pathway	241	52	<0.001
hsa04974	Protein digestion and absorption	150	50	<0.001
hsa00230	Purine metabolism	225	38	0.013
hsa05146	Amoebiasis	115	37	<0.001
hsa01230	Biosynthesis of amino acids	203	33	0.031
hsa02010	ABC transporters	182	31	0.020
hsa04022	cGMP-PKG signaling pathway	177	31	0.014
hsa04072	Phospholipase D signaling pathway	159	29	0.010
hsa04270	Vascular smooth muscle contraction	149	28	0.007
hsa00240	Pyrimidine metabolism	122	25	0.004
hsa05230	Central carbon metabolism in cancer	107	25	0.001
hsa04721	Synaptic vesicle cycle	90	24	<0.001
hsa05032	Morphine addiction	99	24	<0.001
hsa04724	Glutamatergic synapse	122	23	0.014
hsa00480	Glutathione metabolism	95	22	0.001
hsa04978	Mineral absorption	88	21	0.001
hsa05031	Amphetamine addiction	78	19	0.001
hsa04727	GABAergic synapse	98	19	0.018
hsa04918	Thyroid hormone synthesis	96	19	0.015
hsa04916	Melanogenesis	107	19	0.041
hsa04911	Insulin secretion	98	19	0.018
hsa00561	Glycerolipid metabolism	99	18	0.038
hsa00052	Galactose metabolism	77	16	0.015
hsa05133	Pertussis	86	16	0.040
hsa05033	Nicotine addiction	47	15	<0.001
hsa05030	Cocaine addiction	56	15	0.002
hsa00250	Alanine, aspartate and glutamate metabolism	65	14	0.017
hsa01523	Antifolate resistance	48	12	0.008
hsa04964	Proximal tubule bicarbonate reclamation	40	10	0.015
hsa05143	African trypanosomiasis	45	10	0.033

[a]KEGG: Kyoto Encyclopedia of Genes and Genomes.

Supplemental table 3. Demographic and clinical characteristics of ESCC, CRC, lung cancer patients and healthy volunteers in external validation cohort.

Characteristics	ESCC[a]	CRC[e]	Lung cancer	Healthy volunteers	*p* value
Total Number	81	40	41	58	
Gender (male/female)	72/9	31/9	24/17	44/14	0.002
Age (mean ± SD[b], year)	65.38 ± 7.93	60.60 ± 11.16	61.27 ± 9.00	62.38 ± 10.36	0.027
BMI[c] (mean ± SD[b])	22.12 ± 3.00	24.45 ± 3.47	24.45 ± 3.53	23.36 ± 3.68	<0.001
Smoker, n (%)	48 (59.3)	7 (17.5)	16 (39.0)	16 (27.6)	<0.001
Drinker, n (%)	41 (50.6)	4 (10.0)	13 (31.7)	12 (20.7)	<0.001
TNM[d], n (%)					
0-I	13 (16.0)	6 (15.0)	16 (39.0)	—	
II	22 (27.2)	12 (30.0)	2 (4.9)	—	
III	40 (49.4)	16 (40.0)	16 (39.0)	—	
IV	6 (7.4)	6 (15.0)	7 (17.1)	—	

[a]ESCC, esophageal squamous cell carcinoma; [b]SD, standard deviation; [c]BMI, body mass index; [d]TNM, tumor-node-metastasis classification system (8th edition, 2017); [e]CRC: colorectal cancer.

Supplemental table 4. Logistic regression analysis for association between demographic or clinical characteristics and ESCC.

Characteristics	Total (n)	Odds Ratio (95% CI)	*p* value
Age (<60 vs. ≥60)	23	0.86 (0.16-4.55)	0.855
Gender (Male vs. Female)	23	1.13 (0.17-7.70)	0.901
Smoker (NO vs. YES)	23	0.86 (0.16-4.55)	0.855
Drinker (NO vs. YES)	23	1.75 (0.33-9.93)	0.511
BMI[a] (<25 vs. ≥25)	23	1.14 (0.20-6.62)	0.879
TNM[b] (I-II vs. III-IV)	23	13.33 (2.07-130.86)	0.012
Clinical T stage (T1-T2 vs. T3)	23	14.00 (1.80-302.65)	0.028
Clinical N stage (N0 vs. N1-N3)	23	13.33 (2.07-130.86)	0.012
Tumor size (<5 vs. ≥5)	23	29.33 (3.52-675.53)	0.007

[a]BMI, body mass index; [b]TNM, tumor-node-metastasis classification system (8th edition, 2017)

Supplemental table 5. Expression differences of HPRT1 between various types of cancer tissues and corresponding normal tissues from TCGA database.

Abbr	Cancer type	Cancer tissues (n)	Normal tissues (n)	W[a] value	*p* value
LCA	Bladder Urothelial Carcinoma	411	19	6199	<0.001
BRCA	Breast invasive carcinoma	1104	113	105652	<0.001
CESC	Cervical squamous cell carcinoma and endocervical adenocarcinoma	306	3	881	0.006
CHOL	Cholangiocarcinoma	36	9	215.5	0.133
COAD	Colon adenocarcinoma	471	41	11554.5	0.037
ESCA	Esophageal carcinoma	162	11	1682	<0.001
GBM	Glioblastoma multiforme	168	5	43	0.001
HNSC	Head and Neck squamous cell carcinoma	502	44	19350	<0.001
KICH	Kidney Chromophobe	65	24	1346	<0.001
KIRC	Kidney renal clear cell carcinoma	535	72	5077.5	<0.001
KIRP	Kidney renal papillary cell carcinoma	289	32	1781.5	<0.001
LIHC	Liver hepatocellular carcinoma	374	50	10203.5	0.295
LUAD	Lung adenocarcinoma	526	59	27290	<0.001
LUSC	Lung squamous cell carcinoma	501	49	22503	<0.001
PAAD	Pancreatic adenocarcinoma	178	4	489	0.204
PCPG	Pheochromocytoma and Paraganglioma	183	3	496	0.017
PRAD	Prostate adenocarcinoma	499	52	12841	0.903
READ	Rectum adenocarcinoma	167	10	1015	0.254
SARC	Sarcoma	263	2	275	0.915
SKCM	Skin Cutaneous Melanoma	471	1	81.5	0.260
STAD	Stomach adenocarcinoma	375	32	10866	<0.001
THCA	Thyroid carcinoma	510	58	18567	0.001
THYM	Thymoma	119	2	120	0.992
UCEC	Uterine Corpus Endometrial Carcinoma	548	35	10535	0.328

[a]The Wilcoxon test was used to examine the difference in the expression level of HPRT1 between cancer and normal tissues in each cancer type.

Supplemental table 6. Univariate Cox regression analysis on 33 types of cancer from TCGA database.

Abbr	Cancer type	n	HPRT1 expression level (median)	High/low[a]	HR[b] (95% CI[c])	*p* value
ACC	Adrenocortical carcinoma	79	10.86	50/29	3.020 (1.213−7.519)	0.018
BLCA	Bladder Urothelial Carcinoma	406	11.96	41/365	0.559 (0.318−0.985)	0.044
BRCA	Breast invasive carcinoma	1082	11.94	140/942	1.398 (0.928−2.107)	0.109
CESC	Cervical squamous cell carcinoma and endocervical adenocarcinoma	293	11.16	225/68	0.573 (0.347−0.945)	0.029
CHOL	Cholangiocarcinoma	36	9.77	20/16	1.929 (0.728−5.109)	0.186
COAD	Colon adenocarcinoma	448	10.72	221/227	1.390 (0.915−2.111)	0.123
DLBC	Lymphoid Neoplasm Diffuse Large B-cell Lymphoma	47	12.27	5/42	4.309 (0.787−23.586)	0.092
ESCA	Esophageal carcinoma	161	12.48	20/141	2.215 (1.143−4.293)	0.018
GBM	Glioblastoma multiforme	167	10.78	17/150	2.331 (1.341−4.051)	0.003
HNSC	Head and Neck squamous cell carcinoma	501	10.98	312/189	2.240 (1.648−3.044)	<0.001
KICH	Kidney Chromophobe	64	13.01	8/56	4.518 (1.124−18.167)	0.034
KIRC	Kidney renal clear cell carcinoma	531	11.13	66/465	1.541 (1.028−2.312)	0.036
KIRP	Kidney renal papillary cell carcinoma	286	10.45	101/185	3.411 (1.855−6.271)	<0.001
LAML	Acute Myeloid Leukemia	132	9.82	106/26	2.119 (1.091−4.117)	0.027
LGG	Brain Lower Grade Glioma	524	8.91	387/137	1.823 (1.188−2.798)	0.006
LIHC	Liver hepatocellular carcinoma	368	9.72	267/101	1.757 (1.133−2.724)	0.012
LUAD	Lung adenocarcinoma	513	11.38	108/405	1.469 (1.056−2.044)	0.023
LUSC	Lung squamous cell carcinoma	493	12.52	53/440	0.681 (0.428−1.083)	0.105
MESO	Mesothelioma	84	10.4	42/42	2.727 (1.680−4.425)	<0.001
OV	Ovarian serous cystadenocarcinoma	378	12.19	39/339	0.613 (0.378−0.992)	0.046
PAAD	Pancreatic adenocarcinoma	177	10.8	66/111	1.596 (1.058−2.407)	0.026
PCPG	Pheochromocytoma and Paraganglioma	183	10.79	158/25	0.099 (0.022−0.448)	0.003
PRAD	Prostate adenocarcinoma	499	10.47	185/314	3.137 (0.891−11.041)	0.075
READ	Rectum adenocarcinoma	158	9.6	128/30	86446242.025 (0.000−Inf)	0.996
SARC	Sarcoma	260	10.36	150/110	1.809 (1.185−2.760)	0.006
SKCM	Skin Cutaneous Melanoma	457	10.46	287/170	0.655 (0.497−0.864)	0.003
STAD	Stomach adenocarcinoma	350	12.01	40/310	0.557 (0.293−1.059)	0.074
TGCT	Testicular Germ Cell Tumors	139	10.26	67/72	649445146.333 (0.000−Inf)	0.999
THCA	Thyroid carcinoma	509	11.23	90/419	0.000 (0.000−Inf)	0.997

THYM	Thymoma	118	10.54	77/41	0.288 (0.074−1.118)	0.072
UCEC	Uterine Corpus Endometrial Carcinoma	544	10.76	107/437	2.652 (1.703−4.130)	<0.001
UCS	Uterine Carcinosarcoma	54	10.32	35/19	3.986 (1.628−9.757)	0.002
UVM	Uveal Melanoma	80	9.69	28/52	2.663 (1.148−6.179)	0.023

[a]HPRT1 expression value was dichotomized into high and low groups using median. P-value was calculated using univariate cox regression analysis. [b]HR：hazard ratio; [c]CI: confidence interval.

Supplemental table 7. The qPCR primers.

Primer	Sequence (5'→3')
HPRT1-F	TTGCTTTCCTTGGTCAGGCA
HPRT1-R	ATCCAACACTTCGTGGGGTC
GAPDH-F	GCACCGTCAAGGCTGAGAAC
GAPDH-R	TGGTGAAGACGCCAGTGGA

Supplemental figures

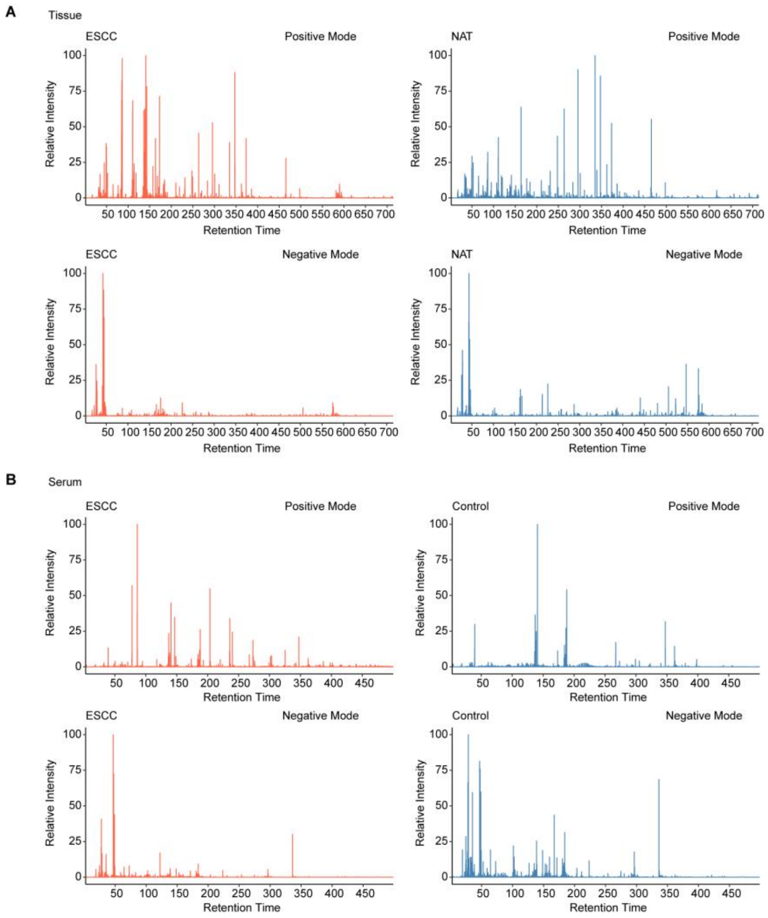

Supplemental Figure 1. The typical Base Peak Chromatograms of tissue and serum samples. (A) Typical base peak chromatograms of the tumor tissues and NAT. (B) Typical base peak chromatograms of the serum samples from patients with ESCC and healthy volunteers. X-axis: retention time; Y-axis: relative intensity of metabolic peaks; red: metabolic peaks detected in positive mode of electron spray ionization; blue: metabolic peaks detected in negative mode of electron spray ionization. ESCC: esophageal squamous cell carcinoma; NAT: normal tissues adjacent to the tumor.

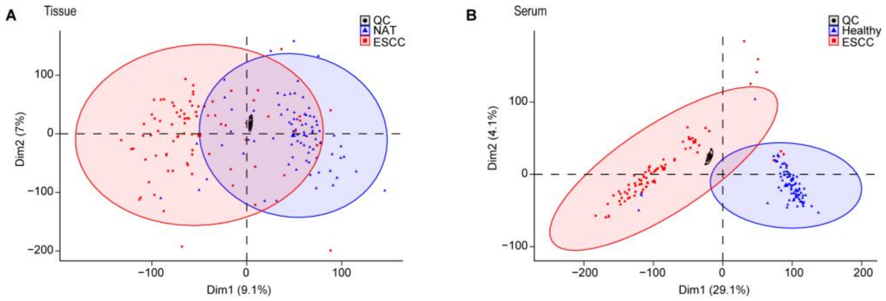

Supplemental Figure 2. Results of PCA analyses for ESCC tissue and serum datasets. (A) The PCA plots of metabolic profiles of tissue discovery set; QC samples are clustered tightly. (B) The PCA plots of metabolic profiles of serum discovery set; QC samples are clustered tightly. ESCC: esophageal squamous cell carcinoma; PCA: principal component analysis; QC: quality control.

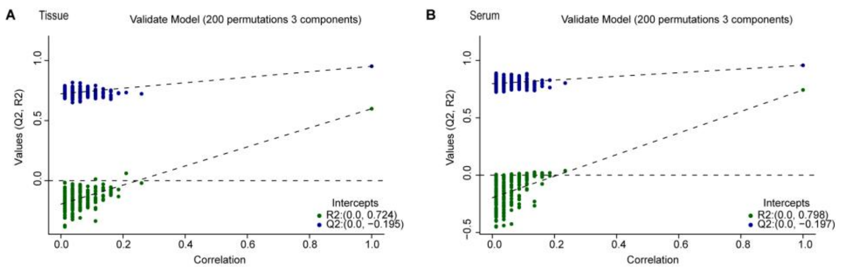

Supplemental Figure 3. Results of permutation tests for PLS-DA models. (A) The permutation test result indicates that the PLS-DA model of tissue discovery set is not over-fitting. (B) The permutation test result indicates that the PLS-DA model of serum discovery set is not over-fitting. PLS-DA: partial least squares–discriminant analysis.

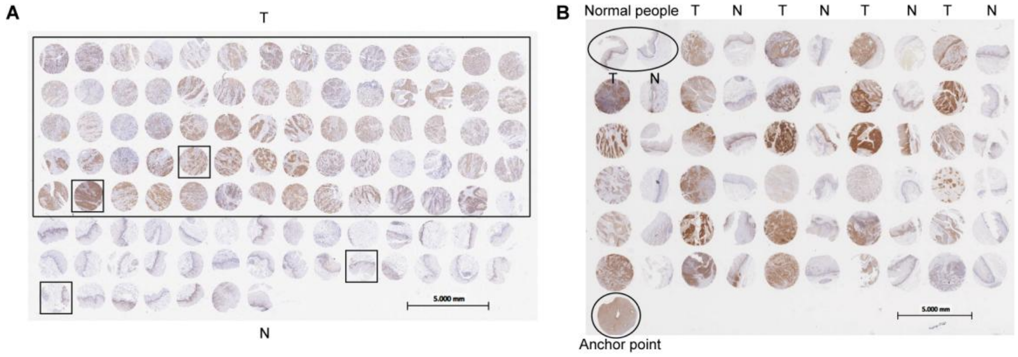

Supplemental Figure 4. Immunohistochemistry on ESCC tissues and NAT using HPRT1 antibody. (A) The no.1 human ESCC tissue microarray consists the tumor tissue specimens (n=35) and corresponding NAT specimens (n=35) from our research cohort. (B) The no.2 human ESCC tissue microarray (purchased from Shanghai Outdo Biotech Co.LTD) consists tumor tissue specimens (n=29) and corresponding NAT specimens (n=29), and the tissue specimens from normal people was excluded in the analysis (n=2). ESCC: esophageal squamous cell carcinoma; NAT: normal tissues adjacent to the tumor.

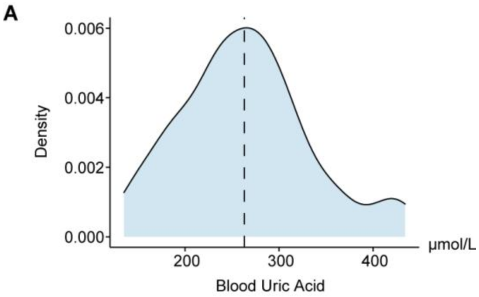

Supplemental Figure 5. Blood uric acid level in patients with ESCC. (A) The distribution of blood uric acid levels in patients with ESCC from discovery cohort (n = 81). ESCC: esophageal squamous cell carcinoma.

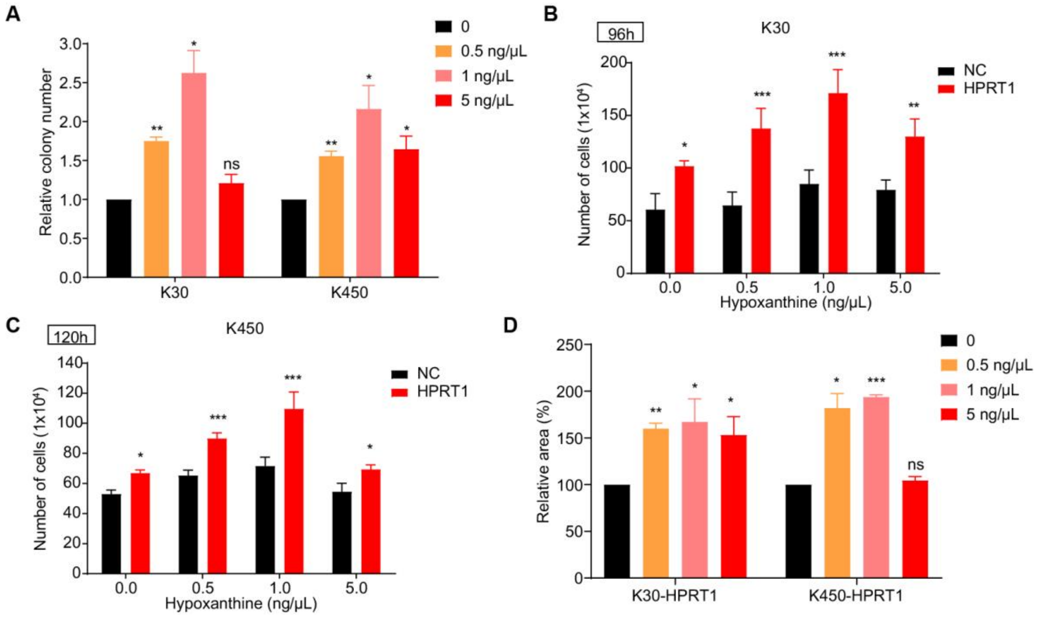

Supplemental Figure 6. Hypoxanthine supplementation combined with HPRT1 overexpression significantly enhances the proliferation of ESCC cells. (A) Hypoxanthine supplementation promoted proliferation of KYSE-30 and KYSE-450 cells. (B-C) Cell proliferation assays indicated that hypoxanthine supplementation combined with HPRT1 overexpression significantly enhanced the proliferation of ESCC cells compared to hypoxanthine supplementation alone. (D) Hypoxanthine stimulated proliferation of HPRT1 overexpressed KYSE-30 and KYSE-450 cells. ESCC: esophageal squamous cell carcinoma. Two-tailed unpaired t test, ns. no significance, *p<0.05, **p<0.01, ***p< 0.001.

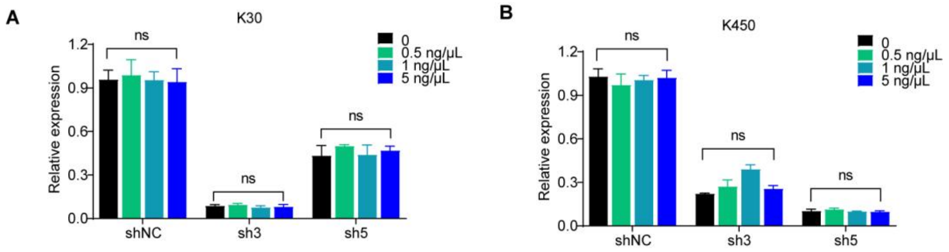

Supplemental Figure 7. The hypoxanthine concentration has no effect on the expression of HPRT1. (A-B) Relative mRNA expression of HPRT1 after culturing ESCC cells with different concentrations of hypoxanthine. Two-way ANOVA test, ns. no significance.

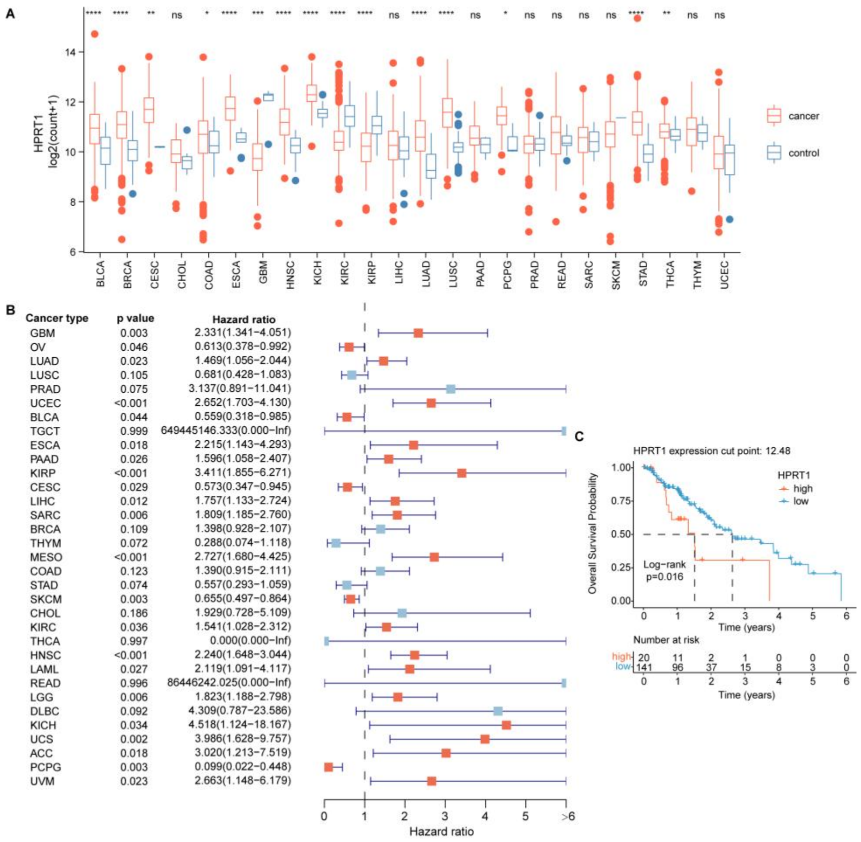

Supplemental Figure 8. HPRT1 expression and its association with prognosis across multiple cancer types. (A) HPRT1 expression between tumor tissues and corresponding normal tissues in 24 tumor types from The Cancer Genome Atlas (TCGA). Wilcoxon rank-sum test, ns. no significance, *p<0.05, **p<0.01, ***p< 0.001, ****p< 0.0001. (B) The results from Cox proportional hazards model showing, in 17 types of cancer, the high HPRT1 expression group having a significantly increased risk of death compared with the low HPRT1 expression group. Especially in esophageal cancer, the HPRT1 high expression group having a more than 2-fold mortality risk compared to that of low expression group (HRs=2.215, 95% CI: 1.143-4.293, p=0.018). (C) Kaplan-Meier survival curve with log-rank test showing that high HPRT1 level correlated with poor overall survival of esophageal cancer patients (p=0.016). ESCC: esophageal squamous cell carcinoma. HR: Hazard Ratio. CI: confidence interval.

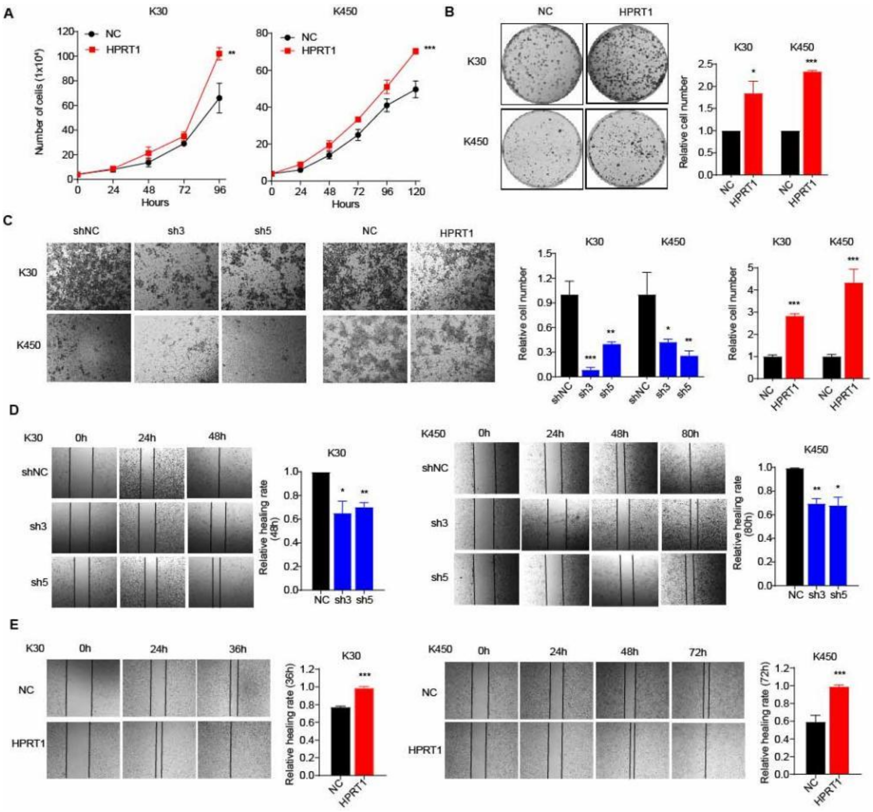

Supplemental Figure 9. HPRT1 promoted malignant proliferation and invasion of ESCC cells in vitro. (A) HPRT1 overexpression promoted the proliferation of ESCC cells. (B) HPRT1 promoted clonogenicity of KYSE-30 and KYSE-450 cells. (C) Representative images and the quantified data from transwell assays performed in KYSE-30 and KYSE-450 cells (scale bar = 100 μm). (D-E) Representative images and the quantified data from wound healing assays performed in KYSE-30 and KYSE-450 cells. Two-tailed unpaired t test, *p<0.05, **p<0.01, ***p< 0.001.
