# Supplementary figures and images for: Purine salvage–associated metabolites as biomarkers for early diagnosis of esophageal squamous cell carcinoma: a diagnostic model–based study
Source: Cell Death Discov. 2024 Mar 14;10:139. doi: 10.1038/s41420-024-01896-6 (PMC10940714; doi:10.1038/s41420-024-01896-6)

Figure 4C

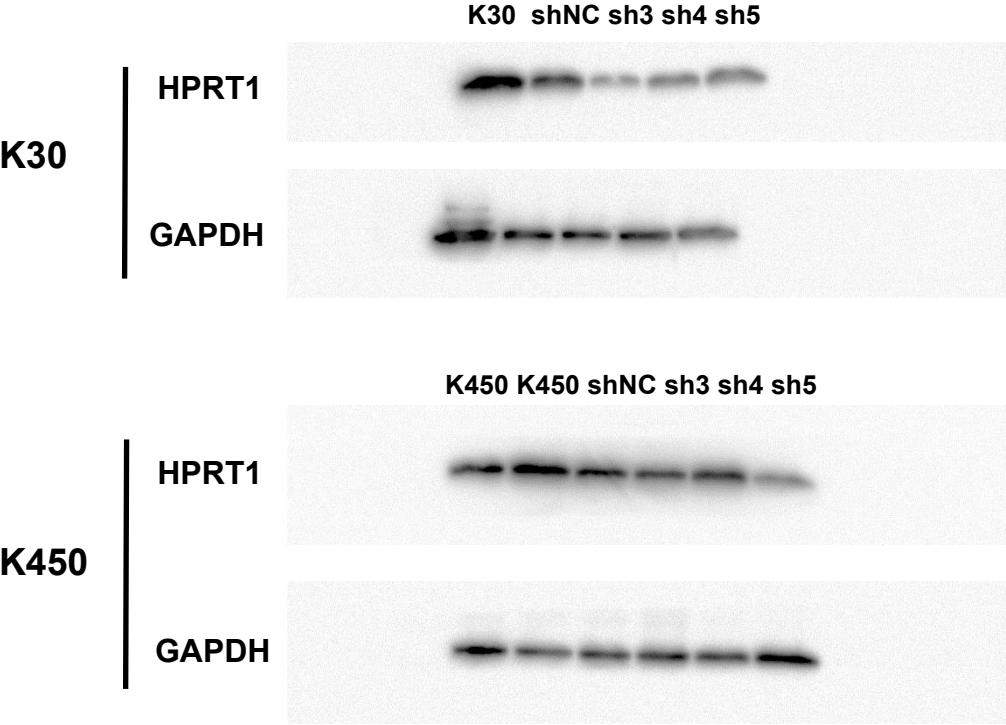

Figure 4D

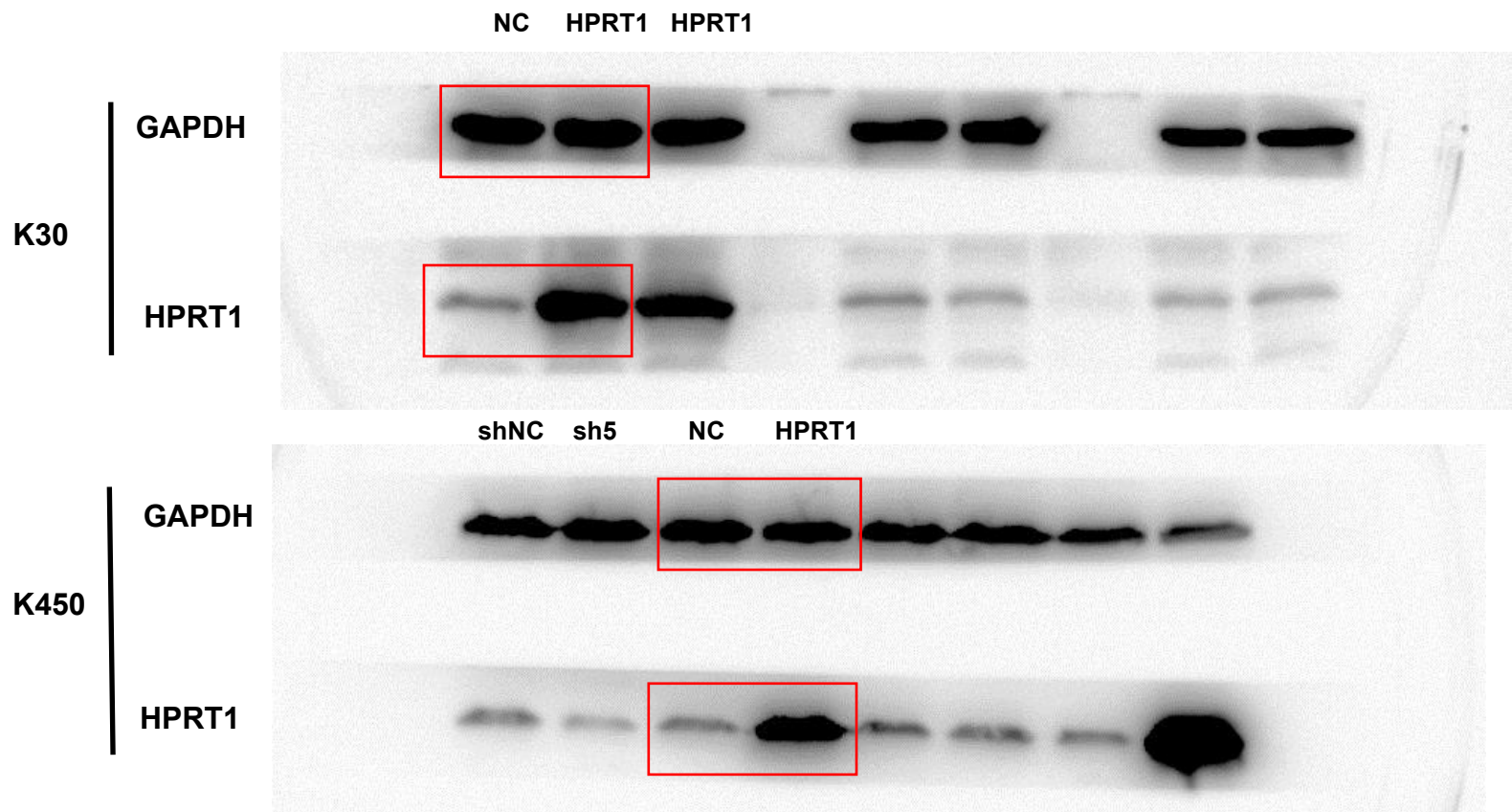

Figure 5G

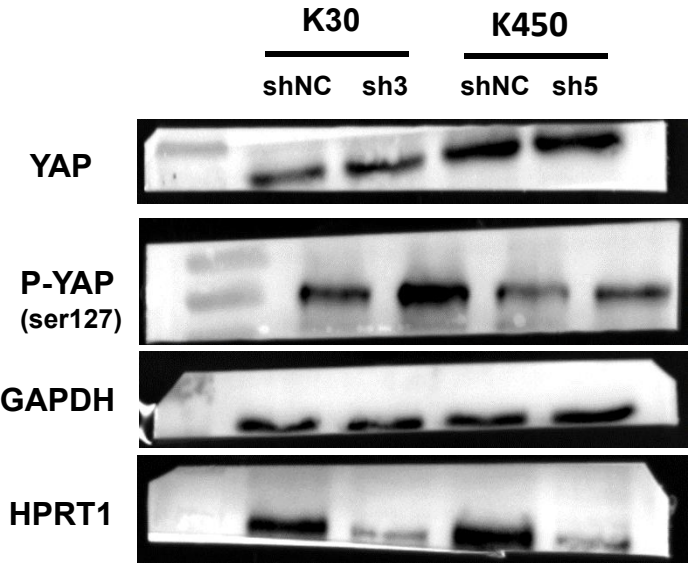

Figure 5H

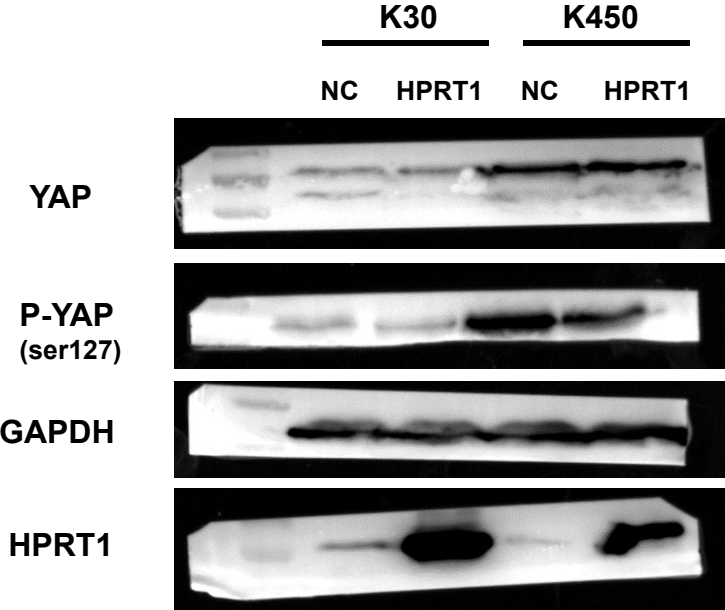

Supplement: Supplementary file 3 — Original western blots [file 41420_2024_1896_MOESM3_ESM.pdf]
